# Architecturally diverse proteins converge on an analogous mechanism to inactivate Uracil-DNA glycosylase

**DOI:** 10.1093/nar/gkt633

**Published:** 2013-07-26

**Authors:** Ambrose R. Cole, Sapir Ofer, Ksenia Ryzhenkova, Gediminas Baltulionis, Peter Hornyak, Renos Savva

**Affiliations:** ^1^Department of Biological Sciences, Institute of Structural and Molecular Biology, Birkbeck College, University of London, Malet Street, London WC1E 7HX, UK and ^2^Research Department of Structural and Molecular Biology, Institute of Structural and Molecular Biology, University College London, Gower Street, London WC1E 6BT, UK

## Abstract

Uracil-DNA glycosylase (UDG) compromises the replication strategies of diverse viruses from unrelated lineages. Virally encoded proteins therefore exist to limit, inhibit or target UDG activity for proteolysis. Viral proteins targeting UDG, such as the bacteriophage proteins ugi, and p56, and the HIV-1 protein Vpr, share no sequence similarity, and are not structurally homologous. Such diversity has hindered identification of known or expected UDG-inhibitory activities in other genomes. The structural basis for UDG inhibition by ugi is well characterized; yet, paradoxically, the structure of the unbound p56 protein is enigmatically unrevealing of its mechanism. To resolve this conundrum, we determined the structure of a p56 dimer bound to UDG. A helix from one of the subunits of p56 occupies the UDG DNA-binding cleft, whereas the dimer interface forms a hydrophobic box to trap a mechanistically important UDG residue. Surprisingly, these p56 inhibitory elements are unexpectedly analogous to features used by ugi despite profound architectural disparity. Contacts from B-DNA to UDG are mimicked by residues of the p56 helix, echoing the role of ugi’s inhibitory beta strand. Using mutagenesis, we propose that DNA mimicry by p56 is a targeting and specificity mechanism supporting tight inhibition via hydrophobic sequestration.

## INTRODUCTION

Interactions between cells and viruses prime the development and acquisition of a multitude of cellular innate immune responses and, reciprocally, anti-restriction strategies. The archetypal examples are restriction-modification systems of prokaryotes as defences against bacteriophages. DNA bacteriophages are found to select against host restriction endonuclease recognition sequences in their genomes (1). DNA phages also use genome-cloaking methods, such as base modification (2–4) or the incorporation of non-canonical DNA nucleotides such as deoxyuridine (5,6), to evade restriction enzyme recognition or cleavage. Although uracilation of DNA may afford protection to viral genomes from restriction endonucleases (3,4), it is nevertheless a prime substrate for the ubiquitous cellular DNA base-excision repair (BER) pathway. Uracil-DNA therefore paradoxically appears to provide no defence against catastrophic disintegration of viral genomes (7). There nonetheless exist viruses, which subvert host nucleotide biosynthesis so that thymidine is replaced entirely by deoxyuridine in the viral genomic DNA, such as the *Bacillus* phage PBS1 (and its clear-plaque isotype PBS2), which survives by encoding an early protein ‘ugi’ that neutralizes the first step of BER by stoichiometric enzyme inhibition (2,5,8–11).

The BER pathway may be considered as a two part process: in the first part, the target base is removed, and the site is primed for repair by creation of a break in the DNA backbone; then, in the second part, DNA repair is enacted. BER is primed to act on a relatively small but significant number of aberrant bases within a genome that may arise at any time, predominantly from ambient cellular processes. Deoxyuridine is such a target for BER, as it may naturally arise by spontaneous deamination of deoxycytidine, which would lead to C:G to T:A transition mutations. However, active incorporation of deoxyuridine during the replication of certain viruses will result in an unusually high concentration of uracil. Under these conditions, uracil-DNA BER promotes DNA double-strand breaks due to the proximity of substrate sites on the paired DNA strands. A viral DNA genome in which all thymidine is supplanted by deoxyuridine would therefore be reduced to a non-viable pool of fragments by the action of BER (7,8).

In eubacteria and most eukaryotes, the archetypal uracil-DNA glycosylase (UDG) is primarily active in uracil-DNA BER. UDG represents family 1 of a superfamily of enzymes with sequence and structural homology in their functional motifs (12–14). UDG is exquisitely selective for uracil bases located in single- or double-stranded DNA, and it selectively removes uracil by cleaving the N-glycosyl linkage between the base and the deoxyribose, leaving behind an abasic site (15). UDG is able to non-specifically bind and scan DNA bases, capturing thymine and uracil due to natural DNA breathing motions that cause them to un-pair and partially emerge from the helical centre of DNA. By a squeezing and pinching deformation of B-DNA structure and concomitant insertion of a loop into the minor groove, UDG is able to exaggerate this breathing motion of DNA. UDG facilitates this by providing a pseudo base pair for a purine base via the residue at the apex of its minor groove intercalation loop. This intercalation also results in the pyrimidine base of the erstwhile pair being flipped out of the helix entirely and prolongs its residency in the UDG concave active site. Thymidine is observed to meet a steric block and would then regress into the DNA helical core. Deoxycytidine is also rejected, whereas deoxyuridine can progress into the catalytic centre of UDG and is efficiently liberated as uracil (16,17) (Supplementary Figure S1). Family 1 UDG is the BER initiating enzyme that is targeted by a stoichiometric active site inhibitor: the ugi protein encoded by uracil-DNA phage PBS1 to preserve the integrity of its uracil-DNA genome (10,11).

**Figure 1.**
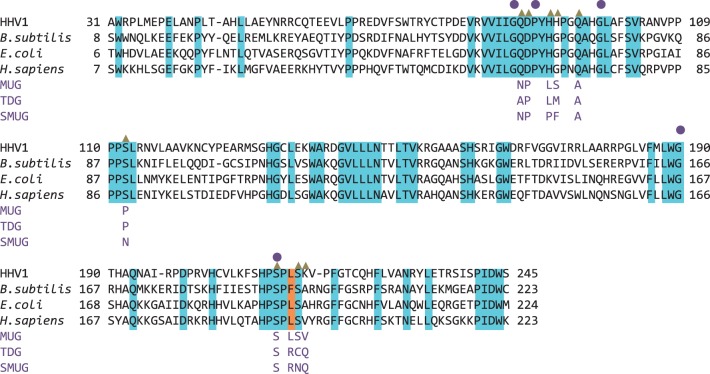
HHV-1, human and *E. coli* UDG are among the most completely characterized UDGs, including structural data of their association with the PBS1 encoded stoichiometric inhibitor, ugi. *Bacillus subtilis* UDG is the natural target of the inhibitors ugi and p56. Sequence identity between these UDGs is highlighted in cyan. Positions marked with a gold triangle show important points of contact between HHV-1 UDG and p56 observed in this study, which are also seen in contacts between distorted B-DNA with human UDG-DNA in structure 1SSP (Figure 3, and Supplementary Table S1). MUG (*E. coli*), TDG (human) and SMUG (*Xenopus*) are other enzymes from the wider UDG superfamily, but important residues in ugi and p56 inhibition are not conserved, these are indicated by aligning only these residues; those residues that are conserved between these enzymes and family 1 UDGs shown, are indicated by a circle above. The orange highlighted region is the UDG minor groove intercalation loop apical hydrophobic residue (phenylalanine in *B. subtilis*, leucine in other UDGs shown) sequestered by ugi and p56. The presence of an arginine at this position in SMUG and TDG suggests lack of susceptibility to ugi or p56 (see main text). Residue substitutions across the much more highly conserved family 1 UDGs can affect attributes such as turnover and dissociation kinetics, and therefore might also modulate interactions with inhibitor proteins.

Branches 2–7 of the UDG superfamily are characterized by their close structural and functional homology or analogy and may be identified and grouped by their occurrence in the domains of life and the particular patterns of motif conservation or variation (12–14). Metazoan genomes may encode more than one type of uracil-DNA BER activity, of which some may be active at different times of the cell cycle or in different tissue types or cellular processes (18). Superfamily alternatives to UDG in eubacteria are ancillary and generally less active uracil-DNA BER activators, which can however deal with bulkier base adducts (19). The UDG superfamily is also represented in extremophiles, as well as in some viruses of disparate lineages (12–14,20,21). In studies of other branches of the UDG superfamily, there are indications that ugi is at least partially inhibitory towards uracil-DNA repair activities other than family 1 UDG, when mixed *in vitro* with cell lysates of relevant organisms (20). It has also been reported that certain branches of the UDG superfamily appear to be unaffected by ugi, the PBS1 phage inhibitor that targets family 1 UDG. However, considering the striking conservation of DNA-binding site structure, this is most likely attributable to residue variations in conserved motifs (19,21) (Figure 1).

Interestingly, some of the phages reported to encode UDG-inhibitory activities do not replace thymine with uracil in their genomic DNA and are instead thought to inhibit UDG to avoid the random occurrence of nicks due to BER in their replicating genomes (22). Salient phages in this category are coliphage T5, and the family of *Bacillus* phages that includes ϕ29, Nf, PZA, B-103 and GA-1. The identity of the UDG inhibitory activity detected on infection by T5 is yet to be confirmed. It is not obvious when considering the reported biophysical properties of the inhibitor, which of several candidate open reading frames (ORFs) in the first 8% of the T5 genome will encode this activity (23). There is no encoded sequence within this region of the T5 genome with obvious sequence similarity either to ugi, or to the protein inhibitors of UDG encoded by the family of *Bacillus* phages that include ϕ29. Indeed, these p56 proteins and the related protein from phage GA-1 show no sequence homology to ugi, and furthermore a reported structure of the free p56 inhibitor displayed no structural homology to ugi (22,24).

The natural target of ugi is *Bacillus subtilis* UDG; however, structural analyses have used various homologues from Human Herpesvirus 1 (HHV-1), *Homo sapiens* and *Escherichia coli*, in which all the characteristic structural features involved in irreversible inhibition are conserved (Figure 1). Meanwhile, mycobacterial UDG homologues are observed to interact with ugi reversibly, largely owing to residue differences in key motifs (25). UDG and ugi form a tight 1:1 stoichiometric complex with an expansive and predominantly polar interface. The UDG DNA-binding cleft is occupied by ugi via stereochemical mimicry of contacts that would otherwise be productively formed by distorted B-DNA (10,11,26). Blockade of the UDG DNA-binding cleft is achieved by an ugi beta strand and is supported by sequestration of the pre-catalytic pseudo base pairing hydrophobic residue at the apex of the UDG DNA minor groove intercalation loop. This arrangement provides exquisite specificity of ugi to UDG, rather than to DNA-binding proteins per se: significant variations in this minor groove intercalating loop, such as in vaccinia virus UDG, are sufficient to confound ugi inhibition (11,21).

Some observations indicate similarities between UDG contacts made by ugi and p56 proteins, respectively. For example, p56 and a relative encoded by phage GA-1, both make essential contacts with the hydrophobic residue on the apex of the UDG minor groove DNA intercalation loop and can be displaced from UDG by ugi implying a similar site of binding (27,28). The p56 protein in its apo-form, however, does not resemble ugi (a monomeric inhibitor of UDG) and, among other differences, features a hydrophobic alpha helical dimer interface, demonstrated by mutagenesis to be indispensible for UDG inhibition (24). Therefore, owing to the radically different architecture of p56 when compared with ugi, inspection of the apo-p56 structure alone cannot lead to a prediction of its mechanism of inhibition of UDG, despite data in support of commonality of interactions.

In the present study, we have sought to address the enigmatic question of the structural basis of ostensibly similar UDG inhibition by entirely unrelated viral proteins. To this end, we have determined the structure of a UDG—p56 complex, and despite the architectural disparity between these two types of inhibitor, we can now observe why the inhibition of UDG, whether imparted by ugi or by p56, is functionally analogous. We find that both types of inhibitor form corresponding interactions with UDG that are surprisingly mutually superimposable and map faithfully to contacts observed in X-ray structures of double-stranded DNA bound to UDG (16). Furthermore, both inhibitor types, in architecturally dissimilar ways, target the UDG DNA minor groove intercalation loop: a key mechanistic component in UDG substrate recognition and catalysis. Using a combination of mutagenesis, equilibration of pre-formed UDG—p56 complexes with free ugi protein, and analysing the effects of free p56 on other members of the UDG superfamily, we propose that p56 DNA mimicry engenders initial targeting and selectivity of UDG: a prelude to tight binding reminiscent of the most avid macromolecular interactions.

We also analyse the convergence on exquisitely selective functional analogy by entirely unrelated UDG inhibitors and discuss its implications for the existence and discovery of other BER modulating proteins at large.

## MATERIALS AND METHODS

TOPO (Invitrogen) plasmids were selected and maintained with 50 µg/ml kanamycin. All other plasmids were selected and maintained with 100 µg/ml ampicillin. LB media were according to Miller. Enzymes and buffers for PCR, DNA modification and cloning were supplied by New England Biolabs (NEB) and used according to manufacturer’s recommendations. Synthetic oligonucleotides were supplied by Eurofins-MWG-Operon. Unless otherwise specified, general reagents were supplied by Sigma-Aldrich. Details of oligonucleotides, genetic sequences and schematic diagrams are in the Supplementary File.

### Cloning of PZA p56 and HHV-1 UDG for bi-cistronic recombinant expression

The protein sequence accession for *Bacillus* phage PZA p56 (GenBank: AAA88480.1) was reverse translated *in silico* with *E. coli* optimized codon usage (29). The synthetic gene (Supplementary File) was supplied in a standard TA-cloning vector by Eurofins-MWG-Operon. HHV-1 UDG was obtained from the construct pTS106.1 (15).

A bi-cistronic cassette was designed (Supplementary File) with p56 as the first ORF, separated from UDG (the second ORF) by a natural intergenic sequence followed by an artificial tag-encoding sequence. The chosen intergenic sequence naturally separates the *E. coli* genes *mopA* and *mopB* (which encode GroEL and GroES). The artificial tag-encoding sequence comprised nucleotides encoding a 10 × His tag, followed by a Strep Tag II, and finally a TEV protease recognition motif. HHV-1 UDG was amplified with a 5′-end encoding from: ‘GGVSP … ’. Synthetic PZA p56, HHV-1 UDG and the *mop* intergenic sequence were amplified by PCR using Phusion DNA polymerase, with primers that defined restriction sites convenient for cloning. The tag-encoding sequence was formed from overlapping oligonucleotides, phosphorylated with T4 polynucleotide kinase and ligated at elevated temperature using *Taq* DNA ligase. The individual amplicons and intact tag sequence were then gel purified from 1.6% agarose, using a QIAquick gel extraction kit (QIAGEN) subsequent to electrophoresis.

The cassette was formed by catenation via overlap extension PCR, which used 2 ng of each of the four purified DNA molecules, with outer primers for p56 (coding strand homology, primer P1), and HHV-1 UDG (reverse strand homology, primer U2). The p56 ORF thus formed the 5′ end of the cassette, followed by the *mop* intergenic region, then by the synthetic tags and finally, HHV-1 UDG formed the 3′ end. The 5′ extremity of the chimera comprised a restriction site for NdeI; the 3′ extremity comprised a HindIII restriction site.

The cassette amplicon was captured into a pCR-Blunt-II-TOPO vector (Invitrogen) and propagated in NEB5α cells (NEB). DNA sequencing (GATC Biotech) indicated 100% sequence identity to that predicted.

A pRSET-C plasmid (Invitrogen) was digested using NdeI and HindIII restriction enzymes, in the presence of CIP alkaline phosphatase and gel purified from 0.8% agarose. The bi-cistronic cassette was released from the latterly described TOPO clone by digestion with NdeI and HindIII restriction enzymes and gel purified from 1% agarose. The plasmid and cassette were ligated using NEB Quick Ligase and propagated in NEB5α cells before final a DNA sequencing check. The plasmid was designated pRSC2056.

### Site-directed mutagenesis and associated construct modification

Oligonucleotides were designed to perform PCR of the pRSC2056 plasmid (Supplementary File). Mutations to p56 created construct modifications E37Q, E37D, Y40N, E37D/Y40N, respectively (designated pRSC2056mn, where n could be Q for E37Q, E for E37D, Y for Y40N or d for E37D/Y40N). Mutations were defined at least 11 nt from the 3′-end on one of a pair of primers; the second primer extended the template in the opposing direction, and there was no overlap and no gap between the 5′-end priming sites of the two primers.

The primers were phosphorylated with T4 polynucleotide kinase before their use in an 18 cycle PCR performed using Phusion DNA polymerase. After PCR, 10 units of DpnI restriction enzyme were added directly to the 50 µl of PCR reactions, and tubes were incubated for 90 min at 37°C. Reaction volumes were then expanded to 100 µl with 1× T4 DNA ligase buffer (NEB) including 400 units of T4 DNA ligase. The tubes were further incubated for 2 h at 30°C, before propagation in NEB5α cells. DNA sequencing was used to identify mutants, indicating mutagenesis efficiencies of 25–100%.

In addition, for the bi-cistronic expression plasmids pRSC2056 and the three p56 mutants E37D, Y40N and E37D/Y40N, a variant construct was also created by deletion of the bulk of the HHV-1 UDG ORF. These plasmids would therefore express only p56 protein (wild-type, or a given mutant, respectively) on induction. The UDG deletion plasmids were designated pRSCΔU56n, where n was any of: W for wild-type, E for E37D, Y for Y40N or d for E37D/Y40N. These constructs were created by separately digesting each plasmid using NheI and XhoI restriction enzymes. The incompatible overhangs were then filled in by incubating 75 ng of each plasmid in 20 µl of reactions with 1× Thermopol buffer (NEB), supplemented with 0.3 mM dNTPs and 4 units Vent_R_® DNA polymerase, incubating at 72°C for 15 min. The reactions were each then expanded to 50 µl with 1× T4 DNA ligase buffer incorporating 400 units T4 DNA ligase and incubated at 30°C for 2 h, before propagation in NEB5α cells. Colony PCR with *Taq* DNA polymerase indicated success rates of >80% per six colonies screened per construct modification reaction. DNA sequencing confirmed the anticipated adjustments.

### Expression and purification of a PZA p56 – HHV-1 UDG complex

High yield recombinant expression of the p56 – UDG complex from the pRSC2056 plasmid was performed in T7 Express *lysY* cells at 37°C in liquid LB including 2% (w/v) α-D-glucose. Scrapings from 8% (v/v) glycerol stocks at −80°C were used to inoculate 5 ml of pre-cultures (one per destination 2 l flask) in liquid LB including 2% (w/v) α-d-glucose, shaken at 37°C for 16 h. Subsequent to harvest at 5000*g*, cell pellets were resuspended in fresh broth as a homogeneous mixture, to seed the expression cultures.

Large-scale growth for expression was performed in 2 l capacity (baffled/fluted) Erlenmeyer flasks, containing 500 ml of liquid LB including 2% (w/v) α-d-glucose. These were inoculated at 1/100 (v/v) from the pre-culture resuspensions then incubated at 37°C with shaking at 220 rpm until the culture reached an absorbance at 600 nm of 0.8–1.0. At this point, each culture was induced by the addition of IPTG to 0.2 mM, followed by 16 h further growth at 37°C.

Cells were harvested by centrifugation at 5000*g*, and pellets were resuspended on ice into 30 ml of ice-cold resuspension buffer per 5 g wet weight of cells. The resuspension buffer was 100 mM Tris–HCl, 200 mM Arginine, 150 mM NaCl, 10% glycerol, 1 mM EDTA, 1 mM PMSF, with a final pH of 7.8. Pellets were immediately frozen at −20°C.

Pellets were partially thawed at 37°C, placed in wet ice and sonicated with three rounds of 6 s pulses over 5 min periods, at 60 W using a Sonics Vibra-cell ultrasonic processor. The lysate was treated with ice-cold streptomycin sulphate [final concentration 1% (w/v)], along with 100 µg egg-white avidin, and then incubated on ice for 30 min. The treated lysate was subsequently centrifuged at 45 000*g* for 50 min in a Beckman Coulter Avanti J-20 XP centrifuge with JA25.50 rotor. The supernatant fraction was then 0.2 µm filtered and applied to a 5 ml StrepTactin superflow cartridge (GE-Healthcare), equilibrated with the same buffer used to resuspend the harvested cells except that PMSF was omitted. Subsequent to sample loading, the cartridge was washed with 10 column volumes of the PMSF-free buffer. Elution used the PMSF-free buffer, supplemented with 2.5 mM desthiobiotin. The peak eluate (typically ∼30 ml) was placed in a 14–16 K cut-off dialysis bag and 0.2 mg TEV protease [prepared from an in-house construct, according to criteria reported elsewhere (30)] was added to the contents. The bag was dialysed with stirring for 12 h at 4°C against 2 l of 20 mM Tris–HCl, 200 mM NaCl at pH 7.8. The dialysed sample was concentrated at 3200*g* to a final volume of 10 ml in a Vivaspin-20 concentrator (Sartorius) with a 10 000 MW cut-off and loaded onto a Superdex 75 HiLoad 26/60 prep grade gel-filtration column (GE-Healthcare). Two distinct peaks were eluted: analysis by Coomassie stained SDS–PAGE revealed that peak one comprised two species consistent with the expected migration characteristics of PZA p56 and HHV-1 UDG, implying their association, whereas peak two was identified by comparative assay as TEV protease and not UDG. Peak one fractions, an apparent complex of PZA p56 and HHV-1 UDG, were concentrated as previously described until the concentration was between 2 and 4 mg/ml then stored at 4°C. Protein thus stored was suitable for crystallization for periods of months under these conditions.

### Expression and purification of PZA p56 mutants co-expressed with HHV-1 UDG

Expression and purification of each of the four p56 mutants was performed exactly as stated in the previous section, concerning the wild-type p56 complex with HHV-1 UDG. The mutant E37Q exhibited purification characteristics indistinguishable from wild-type p56 co-expressed with HHV-1 UDG. However, for the other three mutants, SDS–PAGE analysis revealed that only UDG was present in eluted fractions from StrepTactin, whereas the p56 mutants (E37D, Y40N or E37D/Y40N) were assessed to be in the column flow-through fraction (Supplementary File).

### Expression and purification of apo PZA p56 and apo p56 mutant derivatives

High yield recombinant expression of p56 apo proteins from the pRSCΔU56n plasmids was performed in T7 Express *lysY* cells as described for the pRSC2056 plasmid, with some modifications. Large-scale growth for expression was performed in 0.5 l capacity Erlenmeyer flasks, containing 100 ml capacity liquid LB including 2% (w/v) α-D-glucose. These were inoculated at the equivalent of 1/100 from the pre-culture resuspensions and then incubated at 37°C with shaking at 200 rpm until the culture reached an absorbance at 600 nm of 0.5–0.8. At this point, each culture was induced by the addition of IPTG to 0.2 mM, followed by 16 h further growth at 30°C.

Cells were harvested as described for pRSC2056; however, the resuspension buffer was 20 mM Na-HEPES, 300 mM NaCl with a final pH of 7.5. Pellets were immediately frozen at −20°C. Pellets were lysed as described for pRSC2056; however, incubation with streptomycin sulphate and egg-white avidin was omitted. The filtered supernatant fraction was obtained as described for pRSC2056 and then diluted 6-fold in 20 mM Na-HEPES buffer (pH 7.5) such that NaCl was adjusted to 50 mM, before loading onto a 5 ml Q-sepharose FastFlow cartridge (GE-Healthcare), equilibrated with 20 mM Na-HEPES, 50 mM NaCl (pH 7.5). Subsequent to sample loading, the cartridge was washed with 10–20 column volumes of equilibration buffer until the asymptote closely approached the baseline. Elution was achieved via an isocratic NaCl gradient over 20 column volumes to an end point of 1.6 M NaCl. The peak eluates were obtained at ∼150 mM NaCl and concentrated at 3200*g* to a final volume of 250 µl in a Vivaspin-20 concentrator (Sartorius) with a 3000 MW cut-off and loaded onto a Superdex 75 Tricorn 10/300GL gel-filtration column (GE-Healthcare), pre-equilibrated in the cell resuspension buffer. Peak fractions were re-concentrated, and a second round of gel filtration was performed to obtain essentially homogeneous protein as assessed by Coomassie-stained SDS–PAGE. Proteins were stored at concentrations of 1 mg/ml at 4°C. Proteins were also observed to remain in solution at 4°C in the tens of mg/ml range.

### Gel shifts and activity/inhibition assays

For gel-shift experiments and activity/inhibition assays, all proteins were adjusted by dialysis or SEC, to 20 mM Na-HEPES, 150 mM NaCl (pH 7.8). In addition to the p56 – UDG complex and the wild-type and mutant apo p56 proteins, the following were also prepared: HHV-1 UDG apo protein (15), *E. coli* MUG protein (19) and PBS1 ugi protein (31). Finally, *E. coli* UDG and HsSMUG were obtained from NEB.

All gel shifts were performed by adjusting target protein concentrations such that peak heights of >50 to <200 mAU were observed on passage through a Superdex 75 Tricorn 10/300GL gel-filtration column on an Äkta FPLC system (GE-Healthcare). The column flow rate was set at 1 ml per minute for all runs, and the protein volume entering the column was 200 µl on each run. Protein mixtures were pre-mixed and held on ice for 4 h before column entry. Fraction sizes were set at 0.5 ml and 12 or 13.5% SDS–PAGE stained with Instant Blue (Expedeon), was used to analyse 20 µl each of peak fractions. In the case of p56 mutants Y40N and E37D/Y40N, owing to lower yield and therefore more dilute fractions, up to 100 µl was vacuum evaporated to ∼20 µl before SDS–PAGE analysis. For vacuum concentrated samples, gels were run slowly until the dye front crossed the stacking zone into the resolving gel. This was done to minimize effects arising from the concentrated buffer and salt in such samples; however, banding anomalies and artefacts are nonetheless possible under such conditions.

To monitor enzyme activity and its inhibition, a DNA substrate was prepared by PCR with *Taq* DNA polymerase, using a dNTP mix in which deoxyuridine entirely supplanted thymidine. Reactions were performed at 37°C for 30 min in total volumes of 24 µl [1× NEB buffer #3 for HHV-1 UDG, 1× NEB buffer #1 for HsSMUG, 1× UDG assay buffer (NEB) for *E. coli* UDG] incorporating 5 units of Endonuclease IV (Nfo) (NEB), which was also the case for control DNA samples. Preliminary titration of substrate and enzymes was performed to scout for a level of turnover that could be tracked by agarose gel electrophoresis in the format of a visual ‘wysiwyg’ assay. Agarose gels were 1% (w/v) and contained SYBR® Safe - DNA Gel Stain (Invitrogen). Gels were run with tanks packed in ice with ice-cold 1× TAE buffer, and images were captured with a BioDoc It™ imaging system and Benchtop UV Transilluminator (UVP). HHV-1 UDG was deployed at 3.6 pmol per reaction (150 nM); apo p56 inhibitor was deployed equimolar with UDG, whereas titration of mutants spanned a wide molar excess over UDG from 2-fold to 345-fold. UDG assays were performed with HHV-1 UDG and quality controlled against a commercially obtained *E. coli* UDG (NEB). Owing to a lack of information concerning its molarity, HsSMUG (NEB) assays deployed p56 wild-type at 3.2 µM (77.5 pmol, 1 µg), whereas p56 mutants were deployed at a nominal excess of ∼53.5 µM (1.285 nmol, 16.5 µg). Controls for the visual assay included reactions without Nfo to check for signs of non-specific nuclease activity and microgram quantities of BSA to control for any effects arising from the more excessive relative molar ratios of p56 inhibitor proteins.

### Crystallographic analysis of a PZA p56 – HHV-1 UDG complex

Aliquots of the PZA p56 – HHV-1 UDG complex were transferred to a 10 kDa cut-off Amicon concentrator and spun at 6000*g* until the final concentration was 5.3 mg/ml. At this concentration, the complex was prone to precipitation and had to be used immediately. Crystallization plates for initial screening were prepared using the Mosquito micro volume crystallization robot at both 2:1 and 1:1 ratios of protein to precipitant with volumes of 0.1 µl:0.5 µl and 0.075 µl:0.075 µl. Optimization blocks were prepared using the Multiprobe II liquid handling robot producing 96-well grids varying precipitant concentrations using the four-corner approach to aid robotic setup (32).

From multiple hits in the initial screens, all producing crystals with a similar morphology, two conditions [28 and 90 of the Molecular Dimensions JCSG screen (33)] yielded promising crystals and were further optimized. Condition 28 contained 10% w/v PEG 6 K with 0.1 M HEPES (pH 7), and condition 90 contained 0.1 M Ammonium Acetate 17% PEG 10 K with 0.1 M Bis-Tris (pH 5.5). Crystals grown in an optimized variant of JCSG condition 90 [0.09 M Ammonium Acetate, 14.3% PEG 10K, 0.1 M Bis-Tris (pH 5.5)] displayed the highest quality diffraction. Crystals exhibited a flat rectangular plate morphology with maximum observed dimensions for the largest face around 50 µm by 20 µm. Crystallographic parameters are given in Table 1.

**Table 1.** Crystallographic data collection and refinement statistics

Data collection	
Space group	P2_1_2_1_2_1_
Cell dimensions	
*a*, *b*, *c* (Å)	52.70, 91.38 162.86
α, β, γ (°)	90.0, 90.0, 90.0
Resolution (Å)	81.43-2.13 (2.22-2.13)
*R*_sym_ or *R*_merge_	0.067 (0.572)
*I* / σ*I*	14.3 (2.3)
Completeness (%)	99.2 (99.2)
Redundancy	4.6 (4.6)
Refinement	
Resolution (Å)	2.13
No. reflections	44499
*R*_work_ / *R*_free_	0.171/0.202
No. atoms	
Protein	5212
Ligand/ion	52
Water	409
*B*-factors	
Protein	44.39
Ligand/ion	55.73
Water	46.29
R.M.S. deviations	
Bond lengths (Å)	0.01
Bond angles (°)	1.01
Ramachandran evaluation	Favoured 98.75% Outliers 0.0%
Molprobity score	1.06 100th Percentile

Crystals were flash frozen in liquid nitrogen immediately following their transfer from crystallization droplets into a cryprotectant solution, consisting of the precipitant and 30% ethylene glycol. Cryo X-ray data were collected on beamline IO4-1 at the DIAMOND light source facility. Data sets were collected with a fine slicing methodology using the PILATUS detector (34) and oscillation of 0.15°, exposure of 0.15 s and a transmission of 75% for 1200 images. Initial beam-line processing was performed using the XIA2 pipeline (35,36). Further processing used the programs XDS (37) and AIMLESS (38). Early data set processing revealed a significant anisotropy in the C* direction. Although still present in the final data, anisotropy was acceptably low with the half data set correlation in the C* direction dropping below 0.5 at 2.46 Å.

The structure was successfully phased by molecular replacement with the program PHASER (35,38) using 1 UDG [the apo form of HHV-1 UDG (15)] and 2LE2 [the apo form of ϕ29 p56 (24)], respectively, as search models, searching for two and four copies, respectively. The two copies of UDG located were designated molecules A and B, whereas the four located copies of the p56 monomer were designated molecules C through F. The resulting map contained no clashes and was structurally consistent with a biologically significant complex.

**Figure 2.**
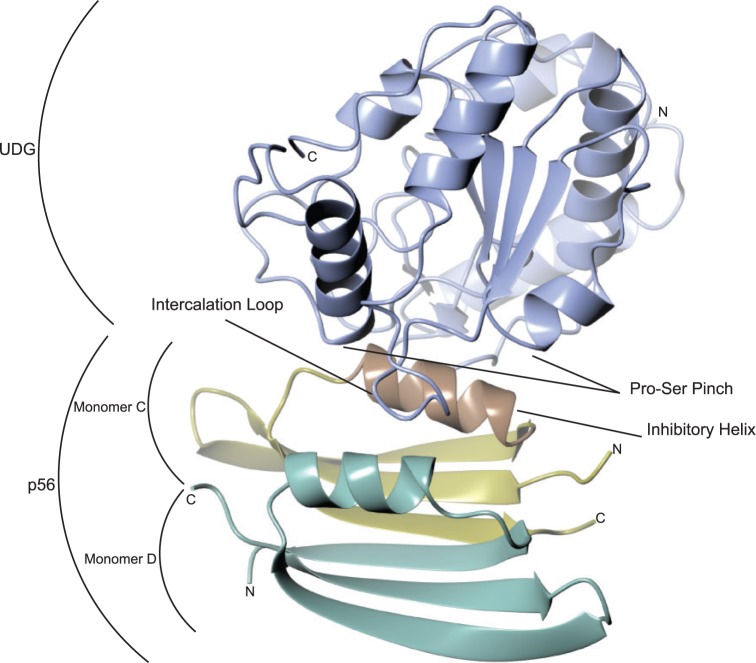
HHV-1 UDG (molecule A, in main text; shown here in light blue) in complex with PZA p56 [PDB accession 4L5N]. The subunits of p56 are coloured to support the descriptions in the text: molecule C (shown here in yellow and brown) makes the most extensive interactions via its docked helix (the brown segment), which includes forming two sides of a hydrophobic pit that traps UDG leucine 214 (see main text for details), whereas molecule D, as well as forming the remaining two sides of the hydrophobic pit, makes just one other notable interaction with UDG (see main text).

Further rounds of refinement used the BUSTER suite of refinement tools (39), and building used the program COOT (35,40). Refinement of the structure yielded high-quality maps for the majority of observed molecules, with the exception of molecule B. Residues 17–60 of molecule B are located adjacent to a large solvent channel in the crystal and poorly ordered in the structure. Omission of the region resulted in clear positive density in fo-fc difference maps but the 2fo-fc maps with the region replaced were consistently poor, especially for side chains. Refinement improved with NCS dropped from this region and TLS refinement introduced with separate operators for the region as well as for each separate chain. Some side chains were still not visible, and these were cropped back to Cβ atoms. The final refinement statistics are shown in Table 1, the overall structure is shown in Figure 2, and the structure is deposited in the PDB with the accession number 4L5N.

Volume calculations were carried out using the analysis program VOIDOO (41) using a probe sphere of 1.4 Å.

## RESULTS

### PZA p56 and HHV-1 UDG form a stable 2:1 stoichiometric complex

Protein p56 of bacteriophage PZA, when co-expressed with HHV-1 UDG in *E. coli*, appeared to form a stable complex. Affinity tag purification of HHV-1 UDG resulted in co-purification of just one other protein, consistent in molecular mass with PZA p56. Dialysis of this material overnight with TEV protease resulted in the expected adjustment in molecular mass of HHV-1 UDG following tag removal, to within 1 kD of the mass of TEV protease (as evidenced using Coomassie SDS–PAGE). Subsequent gel filtration was nevertheless able to resolve HHV-1 UDG from TEV protease, even though the mass difference between these two proteins is <1 kD. This was only possible because HHV-1 UDG co-migrates in a peak some 14 kD larger than anticipated for its apo-form. SDS–PAGE analysis revealed this effect to be due to co-migration with a protein migrating at circa 7 kD. This implies that a dimer of a ∼7 kD protein, consistent with our knowledge of p56, co-migrates with HHV-1 UDG. It is therefore surmised that PZA p56 and HHV-1 UDG remain productively associated from expression through concentration for crystallization.

### Overall structure of the PZA p56:HHV-1 UDG complex

As previously reported, UDG forms a single domain structure consisting of an initial left-handed coil of helices followed by a β−α−β structure that forms an internal four-stranded parallel β-sheet with external α-helices (15). The active site cleft occurs at the end of the sheet where strands 2 and 3 part, with important residues occurring on loops emanating from each of the strands as described previously (15,16). The p56 monomer comprises a three-stranded antiparallel β-sheet with a helix lying along it. The biological dimer is observed to form a six-membered sheet with the helices lying against one another forming a single hydrophobic core (24) (Figure 2).

**Figure 3.**
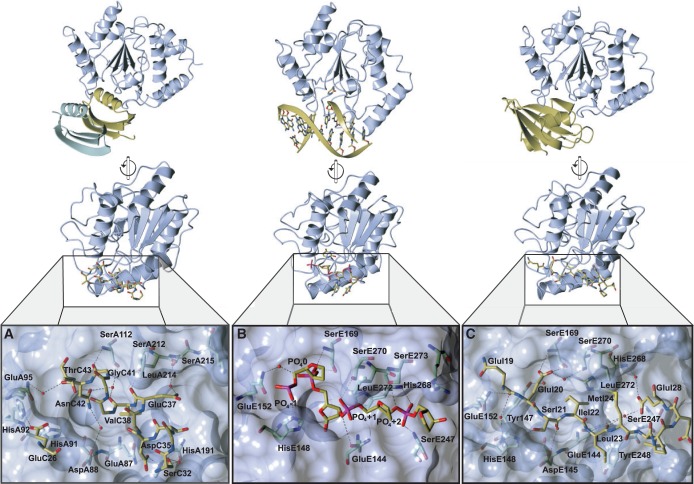
Comparative orientations of UDG bound to p56, DNA, and ugi, with detailed views of the UDG-binding pocket containing both substrate and inhibitor elements on a UDG surface. UDG residues are shown with blue carbon atoms as is the surface, whereas substrate and inhibitor residues are shown with yellow carbon atoms. The second dimer of p56 is shown in green. (**A**) The p56 inhibitory helix bound to the HHV-1 UDG. Only the interacting side chains of the inhibitory helix and Glu26 of p56 are shown alongside UDG interacting residues. (**B**) Backbone of the uracil-containing strand of DNA bound to human UDG (PDB: 1SSP). (**C**) Residues of the ugi inhibitory strand bound to human UDG (PDB: 1UGH).

Most interactions between PZA p56 and HHV-1 UDG in complex (PDB entry 4L5N) are made via the helix of one of the subunits of the inhibitor dimer (molecule C or molecule E, but C only will be referred to henceforth for ease of clarity) (Figure 3a). This p56 helix fits into the UDG DNA-binding cleft (molecule A or molecule B, but only referred to as A henceforth), effectively mimicking DNA (Figures 2b and 3). Supporting this, a small hydrophobic pocket, formed of mirror residues within the helices of the dimer, traps the apical leucine of the HHV-1 UDG DNA minor groove intercalation loop (a phenylalanine in *B. subtilis* UDG), which would normally insert into the DNA helix whenever UDG samples an un-paired base (Figure 5).

### p56 occupies the UDG DNA-binding cleft via an alpha helix

Reminiscent of inhibition by ugi (Figure 3c), the UDG active site is occupied by p56. However, where ugi achieves this blockade with a distorted edge-on beta strand, p56 almost exclusively uses the alpha helix of one of its monomeric subunits (molecule C). Contacts to the UDG DNA-binding cleft from this p56 helix span Ser32 through Asn42. The final contact is via Glu26, which is part of the underlying β-sheets of the inhibitor. Thr43 of the less-associated subunit of the p56 dimer (molecule D; NB: equivalent to molecule F) makes one of only three contacts in total from this subunit (Supplementary Figure S2).

### p56 provides DNA mimetic contacts to UDG analogous to those of ugi

**Figure 4.**
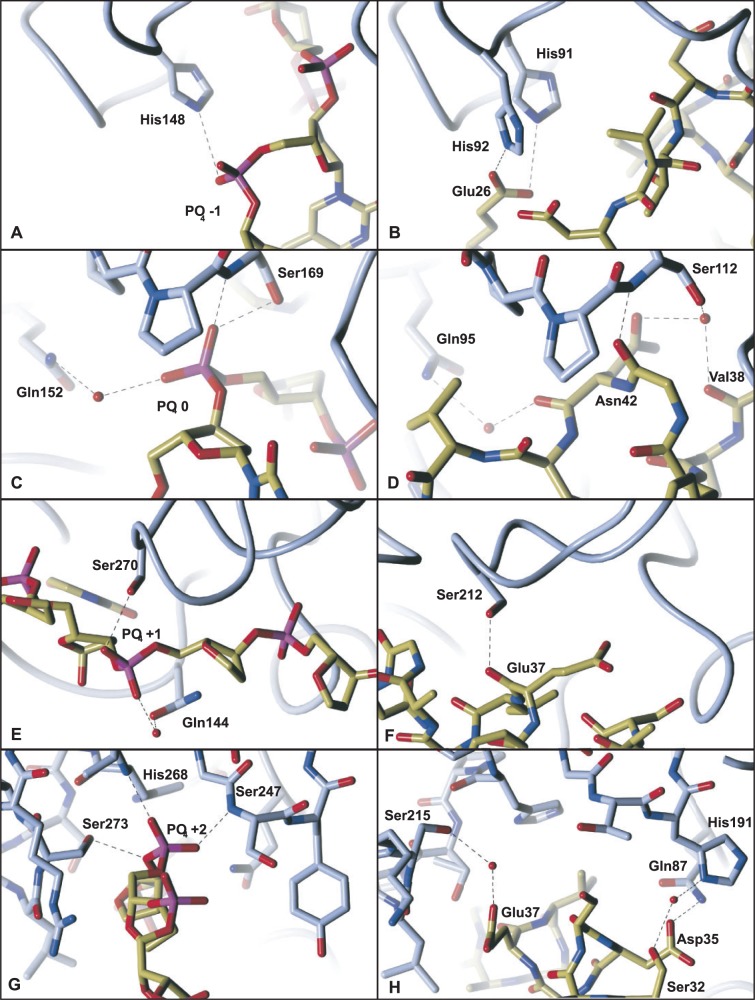
Comparison of UDG-DNA backbone phosphate interactions versus UDG with the inhibitory helix of p56. The UDG carbon atoms are shown in blue, whereas DNA and inhibitor carbon atoms are in yellow. Numbering of UDG residues in left hand side panels (A,C,E,G) refer to human UDG, whereas numbering in right hand side panels (B,D,F,H) refer to HHV-1 UDG; these are equivalent views of the two enzymes. (**A/B**) Area 1 base upstream of the catalysed uracil base compared with the equivalent region with the p56 inhibitor bound. (**C/D**) Region containing the phosphate of the catalysed uracil compared with that with p56 bound. (**E/F**) The phosphate 1 base downstream of the catalysed uracil compared with that with p56 bound. (**G/H**) The final phosphate with significant interactions and the equivalent region with p56 bound.

Many of the HHV-1 UDG interaction points of p56 mirror those made by DNA to UDG, as observed in the structure of human UDG bound to DNA (PDB entry 1SSP) (Figure 3a and b) (16). Several hydroxyl and backbone oxygen interactions closely resemble those formed by the phosphates of the DNA backbone in the kinked conformation exhibited when the uracil base is flipped out (Figure 4). Most residues that stabilize phosphates in the DNA-bound structure have an inhibitor residue interacting with it either directly or via a water moiety (Supplementary Table S1). In comparison, ugi forms a similar number of interactions via its DNA mimicking β-strand (Figure 3c) (10,11). Ugi has an extended interaction with additional points of contact to UDG, and several interactions peripheral to the inhibitory strand, relative to p56 (with reference to the HHV-1 UDG complex). The combination of these factors may contribute to ugi’s reported ability to displace p56 from UDG (22).

### p56 DNA mimicry at position −1 relative to the UDG catalytic pocket

The phosphate before that of the flipped out base (phosphate −1) makes polar contacts with His148 in the 1SSP structure (Figure 4a); this is mimicked in the p56 bound structure by interactions with p56 Glu26 a residue from the underlying sheets rather than the helix occupying the UDG DNA binding cleft (Figure 4b). HHV-1 UDG contains two histidine residues in this region, and both appear in close proximity to the inhibitor residue.

### p56 DNA mimicry at the UDG catalytic pocket and at relative position +1

In the human UDG complex with DNA, the phosphate of the catalysed base (phosphate 0) makes multiple interactions with the N and Oγ of Ser169 (in the Serine-Proline loop that is important in the pinching mechanism of the enzyme) as well as long-range interactions via a water molecule to Gln152 (Figure 4c). These interactions are faithfully repeated in the p56 bound structure with the UDG serine residue NH interacting with the backbone oxygen of p56 Gly41, and the UDG serine OH forming a tight hydrogen bond network via a water with p56 Asn42 Oδ1 and the backbone oxygen of p56 Val38. The DNA interaction with a UDG glutamine is also mimicked through p56 Asn41 O, again via water (Figure 4d). The phosphate following this interacts with Ser270 (human UDG) of the intercalation loop as well as Gln144 (Figure 4e). The bond with the corresponding HHV-1 UDG serine is again mimicked in the inhibitor structure via Glu37 of the inhibitor (Figure 4f).

### p56 mimicry at position +2 relative to the UDG catalytic pocket

The phosphate at position +2 from the catalysed base is the final point of DNA mimicry in the HHV-1 complex with p56. This interacts with (human UDG) Ser273 on the other side of the intercalation loop as well as backbone nitrogen atoms of loops on the other side of the binding cleft (Figure 4g). In the HHV-1 UDG – p56 complex, this is to some extent the exception to superimposable DNA mimicry, as interactions from p56 are noticeably different. Although Ser215 of the UDG DNA minor groove intercalation loop, and p56 Glu37 Oε1 do interact via water, Thr190 (counterpart to serine 247 human UDG) has no close connection. This paucity of interaction may be partly compensated by nearby contacts that are not DNA mimetic, such as the next UDG residue, His191, via a water molecule from p56 Ser32, whereas the side chain Nε2 of UDG Gln87 is seen to bond with Asp35. Finally, Ser34 of the p56 inhibitor could potentially interact but exhibits the wrong geometry in our structure (Figure 4h).

### Analogy in hydrophobic sequestration of a UDG mechanism-specific leucine

**Figure 5.**
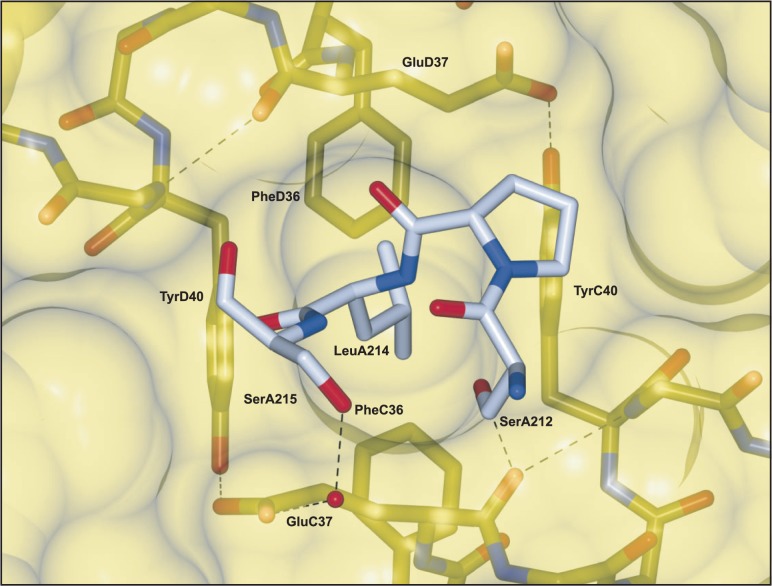
The ‘box-shaped’ hydrophobic pit of the p56 inhibitor dimer with UDG (mol A) leucine 214 inserted. The surface of p56, as well as important residues, is shown in yellow, whereas the UDG carbon atoms are shown in light blue. UDG (mol A) serines 212, and 215, interact with both charged and polar elements of p56 (mol C) Glu37 at the upper rim of the pit, creating a ‘lid’ (see main text).

The tightest point of interaction in the p56 complex appears to be the minor groove intercalation loop of UDG (Figure 5). The apical leucine residue fits into a deep pit between the two helices of the inhibitor dimer. This box-sided pit is formed by a rigid interaction of mutually perpendicular pairs of residues from each p56 subunit helix. Glu37 and Tyr40 side chains from p56 molecules C and D, form the walls of the box. Two opposing corners of the box are formed by hydrogen bonds between the Tyr40 Oη and the carboxyl Oε2 of Glu37. The remaining corners of the box are formed by main chain helical hydrogen bonds between Glu37 and Gly41. The base of the pit is formed by the rings of 2 phenylalanine residues. Despite the presence of the two carboxyl groups in the top corners of this trap, the leucine residue is favourably buried within the hydrophobic interior. When buried in this way, the two flanking serines of the UDG intercalation loop each form hydrogen bonds from their side chain hydroxyls to either end of Glu37 (molecule C). One of these hydrogen bonds is to the side chain carboxyl of Glu37 via a water molecule and the other is to the main chain oxygen, creating the semblance of a hydrophilic ‘lid’ for the box that could presumably make any egress of the leucine unfavourable.

We sought to test the importance of any lid closure via a p56 E37Q mutant (data not shown), as it is possible that the intermediary water molecule might not be retained for the interaction with serine in UDG. However, there still exists a possibility that this water molecule is present because when we view the 3 Å structure of the E37Q p56 complex with HHV-1 UDG, we do not see any significant main chain or side chain rearrangements in this area relative to the wild-type complex (within the margins of error at this resolution), but neither is this a suitable resolution to confidently place a water molecule. Given the apparent structural parity, one could easily speculate that the glutamine head group might rotate 180° (relative to the wild-type configuration) to satisfy interactions with the p56 Tyr40 hydroxyl and the water bridge to UDG serine.

Regardless of the relative importance of the polar lid, however, there is once again analogy with ugi in p56 sequestration of this apical leucine residue in a UDG complex. Using the program Voidoo (41), we calculate that ugi sequesters HHV-1 UDG leucine in a relatively broader hydrophobic well of 149.3 Å^3^, where there appears to be more space particularly near the rim in comparison with the narrow slot of only 86.4 Å^3^ created by the hydrophobic box of p56. Leucine 214 (and its equivalents in other UDGs) adopts different conformations in the various structures available for analysis. In the apo form of HHV-1 UDG, Leu214 adopts a conformation similar to that observed in the described complex with p56, with a shift of 0.5 Å in Cα position but with a similar sidechain conformation; this conformation is also similar in apo human UDG. The ugi bound structure of HHV-1 UDG has a similar Cα position with just 0.4 Å difference, but the opposite leucine conformer is adopted. This conformer is the same in ugi sequestered human, and *E. coli*, UDGs and is therefore likely attributable to a small shift in the position of the hydrophobic trap in ugi relative to p56. It is notable that sequestration of the UDG leucine is a UDG-specific binding feature supporting DNA mimicry that is already contoured to the particular distortions imposed on B-DNA by UDG during binding. This remarkable convergence on an exquisitely UDG-specific mechanism by both p56 and ugi is both unexpected and surprisingly analogous considering the structural disparity between the two types of inhibitor and their converse functional architecture.

### Mutations underline the central importance of the p56 hydrophobic trap

The results of gel shift experiments and inhibitor titrations into enzyme reactions point to the central importance of the p56 box-shaped hydrophobic trap. We designed the mutations with a notion that they would distort the geometry and internal volume of the hydrophobic trap such that binding of the apical hydrophobic residue of the UDG minor groove DNA intercalation loop would be compromised while also aiming to retain the dimeric structure of p56.

Our results would imply that the p56 mutation E37D does not disrupt the dimer interface because the apo protein appears to behave like wild-type p56 in gel filtration chromatography. We also observe, however, that p56 E37D has a reduced interaction with HHV-1 UDG: this p56 mutant does not co-migrate in affinity chromatography following co-expression with affinity tagged UDG (see overloaded sample on SDS–PAGE in Supplementary File), though it does retain an ability to co-migrate with UDG through gel filtration (when both proteins have been separately pre-purified and mixed) (Table 2; also Supplementary File); yet, it does not block UDG activity even at a molar ratio of 50:1 but does so imperfectly (versus a control of an equimolar complex of HHV-1 UDG and wild-type 56) at 100:1 (Table 2; also Supplementary File). Clearly, the indications are that these proteins associate, but not particularly efficiently. Simulation based on the experimental parameters would imply that productive complexes are in the region of 10% (Supplementary Figure S3) (42).

**Table 2.** A summary of data obtained for mutants of p56 and their interactions with HHV-1 UDG

PZA p56 protein variant	Tagged HHV-1 UDG affinity co-purification?	Dimer interface intact?	Migration with HHV-1 UDG on gel filtration?	Inhibition of U-DNA degradation by HHV-1 UDG?
Wild-type	Y	Y	Y	Y
E37Q	Y	Y	Y	Not measured
E37D	N	Y	Partial	Partial^a^
Y40N	N	Impaired	N (?)	N
E37D/Y40N	N	Impaired	N (?)	N

Transit volumes in gel filtration are indicative and supported by SDS–PAGE analysis of fractions across the peak areas (Supplementary File). Degradation of a 100% uracil-DNA substrate can be graded as apparently complete or else inhibition may be seen as DNA being protected partially or apparently completely.

^a^E37D can inhibit HHV-1 degradation of uracil-DNA, but not completely when compared with a sample in which wild-type p56 is present.

(?) The small shoulder on the gel filtration trace (Supplementary File) close to the migration time for the HHV-1 UDG – p56 wild-type complex may be an aggregation product of the p56 mutant in question. SDS–PAGE analysis of overloaded fractions from this part of the trace shows only a band for p56, and not for UDG.

In the case of the Y40N p56 mutation, the situation is complicated by the fact that the dimer interface also appears to be compromised. On gel filtration, there is a distinct two-peak trace, which could represent an aggregated form of the mutant protein (transit time similar to the complex between wild-type p56 and HHV-1 UDG, but no band for UDG visible on overloaded SDS-PAGE sample lanes) and what could plausibly be the monomer (evidenced by indistinguishable banding relative to that seen in the dimer peak on SDS–PAGE): relative peak heights would indicate the monomeric form is predominant. Although co-migration (at a low proportion) of pre-mixed HHV-1 UDG and p56 (Y40N) in gel filtration cannot be ruled out, it should be born in mind that other data for this mutant do not support this. In assay, there is no inhibition of UDG detectable up to 345-fold molar excess. In keeping with these observations, Y40N does not co-purify with affinity tagged HHV-1 UDG from co-expression lysates and its yield following purification is markedly lower than either wild-type or E37D p56. The double mutant (E37D/Y40N) displays similar properties to the Y40N single mutant and is also apparently ineffective in blocking UDG activity; however, owing to a markedly lower yield post-purification, the upper limit of this mutant form of p56 for inhibition assays was a 65-fold molar excess over HHV-1 UDG. This mutant also does not co-purify in affinity chromatography with co-expressed affinity tagged HHV-1 UDG. These data would suggest that the dimer interface is significantly compromised in the Y40N containing mutants.

Taken together, the data from E37D, Y40N and E37D/Y40N mutations of p56 underline the importance of p56 targeting a single UDG residue via a hydrophobic trap. When the mutant data are considered along with behaviour of p56 in competition with ugi, as detailed in the next section, this hydrophobic interaction would appear to be superior in avidity to the supporting DNA mimetic interactions, which are largely water mediated.

### p56 specifically targets family 1 UDG

It is distinctly possible that as well as suggestions of charge-mediated alignment of p56 with UDG (24) as previously suggested for binding of UDG by ugi (11), the DNA mimetic helix of p56 might be more important for specificity of targeting. This can be tested in part by answering the question of whether p56 can inhibit other classes of the UDG superfamily, which have highly conserved active site architecture and geometry, albeit with residue differences (12,18,19). To investigate this, we performed gel filtration analysis of wild-type p56 with *E. coli* MUG and also a visual inhibition assay with human SMUG. These experiments indicated that p56 did not associate with either enzyme (Supplementary File), which is in accord with reports of previous observations involving ugi (19).

Perusal of the structures of *E. coli* MUG and *Xenopus* SMUG1 with superimposition onto our p56 complex to model the possibility of p56 binding reveals that key structure and residue differences could explain the observed lack of inhibition by p56. The critical points for comparison would be the HHV-1 UDG leucine 214 position and its MUG or SMUG equivalents, as well as points along the trajectory of contact of p56 (Figure 1).

With MUG, the structural reasons for lack of interaction with p56 are clearest. The MUG structure is generally more closed around the active site and the UDG Leu214 equivalent position would be less accessible. At the equivalent of phosphate position −1 (phosphate positions are described in Figure 3, and also in Supplementary Table S1) the loop in UDG is a helix in MUG, and there are no equivalences. With respect to p56, MUG residues at the remaining equivalent DNA phosphate positions are either obstructed, spatially removed or substituted and oriented wrongly for interaction.

In the case of SMUG, the most telling substitution is an arginine in the place of the p56-targeted UDG leucine (or phenylalanine in the *B. subtilis* structure). Arginine at this point in *Vaccinia* UDG is known to be sufficient to prevent ugi from blocking UDG activity (11,21). If this SMUG arginine alone would not be convincing enough a reason for lack of inhibition by p56, then there is also the fact that the SMUG DNA-binding cleft positions are not entirely favourable for p56 binding. Although some points along this path are well conserved, e.g. conserved serines at phosphate positions 0 and +1 in both UDG and SMUG, other key interactions are missing or else substitutions would be unfavourable for interaction with p56. Lastly, there is no equivalent to the minor groove loop interactions; therefore, interaction is unlikely with p56. These p56 incompatible features are also seen with another UDG superfamily enzyme, TDG, which has been included in the comparison in Figure 1.

Ugi features more extensive and tighter interactions with HHV-1 UDG than we have observed with p56, and furthermore it is able to displace p56 from UDG (27,28). We ran a crude ugi displacement experiment of p56 co-purified as a complex with HHV-1 UDG. Following overnight rotary incubation at 4°C at microgram per millilitre concentration constituent of 2 mg each of pure ugi (StrepTag II tagged to allow it to be readily distinguished from p56) and the pure HHV-1 UDG – PZA p56 complex, we observed total displacement of p56 by ugi. We noted that an estimated >80% of liberated p56 precipitated during the course of this exchange. However, ordinarily in ambient solution in the buffer that this experiment was performed, p56 is stable up to tens of milligrams per millilitre concentration. This is suggestive of some degree of structural destabilization during the course of displacement of p56. This is potentially due to the hydrophobic effects possible during exchange and would make this an interesting system to investigate in greater depth.

## DISCUSSION

We have observed inhibition of UDG by p56 to be functionally analogous to UDG inhibition by ugi. The most remarkable features of this convergent mechanism of inhibition are that it is imparted by entirely unrelated proteins and uses fundamentally different inhibitor protein architecture in each case. Despite this, in their interactions with UDG both inhibitors successfully mimic stereochemical contacts consistent with B-DNA in the distorted form imposed by UDG binding. In the case of ugi, this is from a β-strand, but we observe that the p56 dimer recruits an α-helix to this role.

Furthermore, this UDG-specific ‘DNA mimicry’ is supported in both inhibitors by the sequestration of a leucine residue (phenylalanine, in the natural target of both inhibitors, *B. subtilis* UDG) at the apex of a loop used by UDG to insert into the DNA minor groove during substrate recognition and catalysis. This contact in p56 may well represent the actual mode of inhibition of UDG and at least appears necessary to provide stability to what is otherwise a highly polar and largely water-mediated set of contacts to the UDG DNA-binding cleft. The extent of these polar contacts are also relatively fewer, and in places more tenuous, in p56 when compared with ugi (with reference to inhibitor complexes involving HHV-1 UDG). The targeted UDG hydrophobic residue is also, however, exquisitely specific to UDG functionality. Both p56 and ugi are seen to use hydrophobic pits to trap this apical loop leucine (phenylalanine in the *B. subtilis* natural target of p56 and ugi); however, again the altogether different structures confer different features. The flexibility of the UDG intercalation loop gives rise to different leucine conformers in the p56 and ugi complexes with HHV-1 UDG. This leucine conformer is at least consistent across different UDGs in complex with ugi (10,11) and therefore probably simply represents the most favourable positioning, given the slight offset between relative positions to UDG of the hydrophobic traps of p56 and ugi.

In the case of ugi, which acts as a monomer when inhibiting UDG, the hydrophobic trap is relatively more spatious, whereas in p56, it is narrow and comprised of essential dimer interface residues. The structure of the p56 hydrophobic pit resembles a narrow open box, with its diametric corners comprised of glutamate head groups contacting tyrosyl hydroxyls. It is plausible that on its way into this ‘box’, the UDG leucine is ‘forced’ past this charged rim by superior forces that facilitate stable docking of p56 to UDG. However, once inside it could be trapped by a combination of the charged rim, above, and the favourable environment, within. Finally, serine residues flanking the leucine on the UDG minor groove DNA intercalation loop effectively seal it inside by creating a ‘lid’ to the box via their interactions with the charged corners of the rim. However, the relative importance of these polar interactions could not be ascertained by perusal of a 3 Å structure of a p56 E37Q mutant. Pondering this ‘polar lid over a hydrophobic pocket’ was reminiscent of another analogy in ligand binding by protein structure: the streptavidin tryptophan cluster, supported by polar loop closure over a bound biotin moiety (43). If the hydrophobic traps of p56 and ugi, which we find analogous to such a tight-binding system, are the main inhibitory interaction from these phage inhibitors to UDG, then it would certainly explain their resistance to dissociation even under harsh conditions.

### Relative avidity for UDG of p56 and ugi

Comparatively, it has been demonstrated that ugi can displace a p56 dimer from UDG (28). This might be due in part to the relatively more extensive contacts of the ugi inhibitory β-strand, compared with the p56 α-helix. If ugi is able to initiate contact to UDG complexed with p56, speculatively at the most polar end of the UDG DNA-binding cleft where p56 serine 32 through p56 serine 34 reside, then it is possible that serial displacement of p56 contacts might occur. The ugi peripheral contacts to UDG, which are not a feature of p56 interaction with the HHV-1 enzyme, must also contribute to relative stability of a UDG – ugi complex. The strongest contact of p56 to UDG is according to our data the trapped leucine; yet, if all other contacts are readily displaced by ugi, then the ultimate displacement of p56 from UDG must according to our data also be relatively assured. This would seem to put blockade of the DNA-binding cleft in the frame more as a UDG selectivity feature, rather than an inhibitory feature under physiological conditions. When the sequestration of HHV-1 UDG Leu214 is compromised without apparent perturbation of the dimer interface, as evidenced by our E37D p56 mutant, a large (but perhaps not non-physiological in the throes of a phage infection) molar excess over UDG (imperfect inhibition is discernable at 100:1) is required to block HHV-1 UDG activity on 100% uracilated DNA. We would propose that the wild-type protein in a phage infection would rapidly occupy available UDG DNA-binding clefts, and we surmise that the hydrophobic trap is the actual site of UDG inhibition by p56. In our crude ‘p56 displacement from UDG by ugi’ experiment described in the ‘Results’ section, the ∼20% of displaced p56 that remained in solution appeared to block UDG activity as effectively as p56 prepared as an apo protein. Therefore, any structural perturbation to p56 during forced removal from UDG by ugi must be recoverable in a minority of events.

### UDG sequestration and inhibition beyond p56 and ugi

From a naïve perspective, all uracil-DNA phages must have some means of neutralizing UDG activity. It is also a fact that phages without genomic uracil-DNA content encode p56-type proteins that directly inhibit UDG. With the benefit of our current observations, it is therefore perhaps no longer as striking that there are few ORFs in phage genomic accessions with anything more than limited sequence homology to a UDG inhibitor of either known type. This situation had been especially curious in the cases of the uracil-DNA *Yersinia* phage ϕR1-37 and the canonical DNA containing coliphage T5, which is reported to encode a 10–15 kD protein that limits UDG activity (6,23). The insights from our study would indicate that bacteriophage proteins, and by extrapolation viral proteins in general, can be unique in sequence and structure, yet analogous in function. This is doubtless owing to the evolutionary pressures inherent in viral replication; however, convergence would not appear to be rare in protein–protein interactions in general (44). We might now conjecture that the reported properties of the UDG inhibiting protein induced by phage T5 appear curiously analogous to our E37D mutant of p56. Could the T5 mechanism feature sufficiently effective specific targeting and blockade of UDG without a requirement for tight inhibition?

We should also bear in mind that the vista of neutralization or modulation of UDG activity by viruses also extends beyond the convergent mechanism described in this report. Alternative types of UDG targeting are also known from viruses of eukaryotes, which result in the trafficking and proteolytic degradation of UDG (45). Modulation of UDG activity of any sort has implications for viral genomic integrity in the informational sense. In the case of HIV-1-infected cells, UDG is sequestered for degradation by the viral protein Vpr, possibly because significant uracil content in HIV-1 DNA appears to be an integral part of the early viral replication strategy (46). Uracil in HIV-1 DNA, together with low-level activity of the antiviral cytosine deaminase APOBEC-3G modulated by the HIV-1 Vif protein, may also ensure a potentially beneficial mutagenic pool of viral progeny (47–49).

UDG therefore constitutes a significant barrier against spontaneous cytosine to uracil conversion from being genetically propagated, and its removal results in informational attrition. The fact that PBS1 DNA was found to have a genomic GC content of ∼28% (5) appears to lend some credence to the argument that its genomic GC content is the result of a downward slide due to silencing of uracil-DNA BER (2). However, bacteriophages T5, and the ϕ29 family, all target UDG activity but have higher genomic GC contents of circa 40%. This difference might be accounted for by the fact that PBS1 is also known to subvert nucleotide biosynthesis pathways, actively skewing the balance in favour of deoxyuridine nucleotide formation (2). Such a downward slide in genomic GC content could also be imagined to be the result of inhibition of other repair systems, such as those targeting 8-oxoguanine. However, given the relative frequency of oxidative damage, inhibition of such systems could quickly become catastrophic for the virus through hypermutation effects (47). Finally, other adaptation pressures may act to lower GC content in phages (49). Nonetheless, it would be interesting to assay for the viral inhibition of BER systems targeting DNA lesions other than uracil.

In summary, we have observed remarkable mechanistic convergence, and striking analogy in host enzyme-specific targeting by two structurally disparate inhibitor proteins encoded by unrelated bacteriophages. Other evidence would also suggest that similar functionalities are at large in the diverse universe of viral proteins. With the benefit of this knowledge, it will now be of some interest to embark on detailed *in silico* studies backed up with recombinant assays and structural biology. This could lead to a more detailed description of diversity in UDG inhibitors and could perhaps also reveal inhibitors of other BER activities, via analysis of further putative examples (6,23). The visual BER assay developed for the work described provides a fast and effective readout for this type of effect. Also, considering the wider roles now assigned to UDG beyond DNA repair (48,50), such discoveries would in turn enable studies to investigate whether bacteriophages are the only viruses that directly inhibit UDG by active site blockade. Enriching our knowledge of the sequence and structural signatures underlying UDG inhibition might eventually reveal whether indeed viruses are the only genomes to modulate UDG activity.

## ACCESSION NUMBERS

PDB 4L5N.

## SUPPLEMENTARY DATA

Supplementary Data are available at NAR Online.

Supplementary Data
