# Supplementary material for: Architecturally diverse proteins converge on an analogous mechanism to inactivate Uracil-DNA glycosylase
Source: Nucleic Acids Res. 2013 Jul 26;41(18):8760–75. doi: 10.1093/nar/gkt633 (PMC3794593; doi:10.1093/nar/gkt633)
Supplement: Supplementary Data [file supp_41_18_8760__index.html]

Architecturally diverse proteins converge on an analogous mechanism to inactivate Uracil-DNA glycosylase — Architecturally diverse proteins converge on an analogous mechanism to inactivate Uracil-DNA glycosylase — Supplementary Data 

# Architecturally diverse proteins converge on an analogous mechanism to inactivate Uracil-DNA glycosylase

## 

files

**Files in this Data Supplement:**

- Supplementary Data - pdf file
