# Supplementary material for: Architecturally diverse proteins converge on an analogous mechanism to inactivate Uracil-DNA glycosylase
Source: Nucleic Acids Res. 2013 Jul 26;41(18):8760–75. doi: 10.1093/nar/gkt633 (PMC3794593; doi:10.1093/nar/gkt633)
Supplement: Supplementary Data [file supp_gkt633_nar-01618-h-2013-File007.pdf]

Supplementary information: Part 1 – Methods

Bacillus phage PZA Gene 1B product [p56] (GenBank: AAA88480.1)

```
1 MVQNDFLDSY DVTMLLQDDN GKQYYEYHKG LSLSDFEVLY GNTVDEIIKL RVDKIS
```

Optimised reverse translation for *E.coli* high-yield expression of PZA p56:

```
  1 atggttcaga acgatttcct cgactcctac gacgtgacca tgcttctgca agatgacaac
 61 ggcaaacagt actatgaata ccataaaggc ctctccctga gcgatttcga agtcctgtac
121 ggtaataccg tagatgaaat catcaaactg cgtgttgaca aaatcagcta a
```

HHV-1 UDG sequence:

With tags -

```
  1 MHHHHHHHHH HASNWSHPQF EKGTENLYFQ STGGVSPAAT SAPLDWTTFR RVFLIDDAWR
 61 PLMEPELANP LTAHLLAEYN RRCQTEEVLP PREDVFSWTR YCTPDEVRVV IIGQDPYHHP
121 GQAHGLAFSV RANVPPPPSL RNVLAAVKNC YPEARMSGHG CLEKWARDGV LLLNTTLTVK
181 RGAAASHSRI GWDRFVGGVI RRLAARRPGL VFMLWGTHAQ NAIRPDPRVH CVLKFSHPSP
241 LSKVPFGTCQ HFLVANRYLE TRSISPIDWS V
```

NB: Tags and TEV recognition site are coloured blue in the above sequence.

Following tag cleavage -

```
  1 STGGVSPAAT SAPLDWTTFR RVFLIDDAWR PLMEPELANP LTAHLLAEYN RRCQTEEVLP
 61 PREDVFSWTR YCTPDEVRVV IIGQDPYHHP GQAHGLAFSV RANVPPPPSL RNVLAAVKNC
121 YPEARMSGHG CLEKWARDGV LLLNTTLTVK RGAAASHSRI GWDRFVGGVI RRLAARRPGL
181 VFMLWGTHAQ NAIRPDPRVH CVLKFSHPSP LSKVPFGTCQ HFLVANRYLE TRSISPIDWS
241 V
```

NB: Key residues: D88, H210, L214 are coloured blue, but their place numbering in the above sequences differs from the HHV-1 original structure, which was produced without tags and tag cleavage.
The original structure numbering, which is relative to the placement of the start codon in pTS106.1 [see citation number 8 in the main manuscript] has been retained throughout the manuscript for clarity, and is derived from translation of the below

HHV-1 DNA sequence for UDG:

```
  1 atggatttaa caaacggggg ggtgtcgcct gcggcgacct cggcgcctct ggactggacc
 61 acgtttcggc gtgtgtttct gatcgacgac gcgtggcggc ccctgatgga gcctgagctg
121 gcgaacccct taaccgccca cctcctggcc gaatataatc gtcggtgcca gaccgaagag
181 gtgctgccgc cgcgggagga tgtgttttcg tggactcgtt attgcacccc cgacgaggtg
241 cgcgtggtta tcatcggcca ggacccatat caccaccccg gccaggcgca cggacttgcg
301 tttagcgtgc gcgcgaacgt gccgcctccc ccgagtcttc ggaatgtctt ggcggccgtc
361 aagaactgtt atcccgaggc acggatgagc ggccacggtt gcctggaaaa gtgggcgcgg
421 gacggcgtcc tgttactaaa cacgaccctg accgtcaagc gcggggcggc ggcgtcccac
481 tctagaatcg gttgggaccg tttcgtgggc ggagttatcc gccggttggc cgcgcgccgc
541 cccggcctgg tgtttatgct ctggggcaca cacgcccaga atgccatcag gccggaccct
601 cgggtccatt gcgtcctcaa gttttcgcac ccgtcgcccc tctccaaggt tccgttcgga
661 acctgccagc atttcctcgt ggcgaaccga tacctcgaga cccggtcgat ttcacccatc
721 gactggtcgg tttga
```

Intergenic sequence (occurs naturally between *E.coli* *mopA* and *mopB*):

```
1 TCCGCGCACG ACACTGAACA TACGAATTTA AGGAATAAAG ATAatgca
```

NB: The intergenic sequence is located downstream of PstI at 3’ of p56 ORF.

Encoding DNA sequences for the tags, and TEV protease cleavage site:

 1 atgcatCACC ACCATCACCA TCACCACCAT CATgctagcA ACTGGTCTCA CCCGCAGTTC
61 GAGAAAggta ccGAAAACCT GTACTTCCAG TCCacc

NB: The above synthetic tag array is contiguous with the intergenic sequence. Restriction enzyme sites are in shown lower case. The 10xHis tag encoding sequence is shown in orange, followed by the Strep Tag II sequence shown in black, with the TEV protease recognition site shown in blue. Half the AgeI site is shown at the 3'-end. This AgeI site is completed by the first UDG codon [ggt].

Oligonucleotide primers for assembly of the p56-UDG expression cassette:

NB:Upper case indicates that the sequence is a reverse complement with respect to coding sense, therefore all primers are written 5' to 3'.

Tag and protease synthetic sequence [with additional 5' homology to intergenic sequence, and additional 3' homology to HHV-1 UDG] -

1 catacgaatttaaggaataaagataa
2 TGGTGATGGTGGTGATGCATTATCTTTATTCCTTAAATTCGTATG
3 tcaccaccatcaccatcaccaccatcatgc
4 TGAGACCAGTTGCTAGCATGATGGTGGTGA
5 tagcaactggtctcacccgcagttcgag
6 GGTTTTCGGTACCTTTCTCGAACTGCGGG
7 aaaggtaccgaaaacctgtacttccagtcca
8 GCAGGCGACACCCCACCGGTGGACTGGAAGTACA
9 ccggtggggtgtcgcctgc

NB: These primers were assembled into a covalent polynucleotide duplex, as described in the methods section of the manuscript.

Oligos for PCR of synthetic PZA p56 ORF [with additional 3' homology to intergenic sequence]:

P1 catatggttcagaacgatttcctc
P2 GTTCAGTGTCGTGCGCGGACTGCAGTTAGCTGATTTTGTCAACACG

NB: 5' NdeI site incorporates ATG

Oligos for PCR of HHV-1 UDG ORF [encoding GGVSP… WSV]:

U1 ggtggggtgtcgcctgc
U2 AAGCTTAAACCGACCAGTCGATGGG

NB: 3' HindIII site incorporates TAA (silent mutations in design - first glycine is ggt from ggg, and stop codon is taa from tga, compared to HHV-1 natural sequence).

Following overlap extension PCR (see manuscript methods), P1 and U2 primed final PCR.

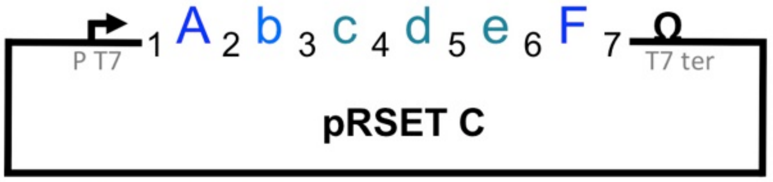

A	PZA p56 synthetic ORF	1	NdeI
b	*mop* intergenic sequence [*E.coli*]	2	PstI
c	10 x His tag	3	NsiI
d	Strep Tag II	4	NheI
e	TEV protease recognition sequence	5	KpnI
F	HHV1 UDG ORF	6	AgeI
		7	HindIII

Schematic of the bi-cistronic expression construct pRSC2056 – with summarised detail of the p56-UDG cassette.

Primers for mutagenesis of p56:

Reverse oligos, point mutations as specified.
M1 [E37D] `GTCGAAATCGCTCAGGGAG`
M2 [E37Q] `CTGGAAATCGCTCAGGGAG`
M3 [Y40N] `GTTCAGGACTTCGAAATCG`

Forward oligo for construction of E37 mutants:
M4 [E37D|Q] `gtcctgtacggtaataccg`

Forward oligo to simultaneously make E37 double mutants with Y40N
M5 [E37D|Q / Y40N] `gtcctgaacggtaataccgtag`

Forward oligo for construction of Y40N single mutant
M6 [Y40N] `ggtaataccgtagatgaaatc`

Deletion of HHV-1 UDG region from NheI to XhoI (near 3'-end of UDG ORF)

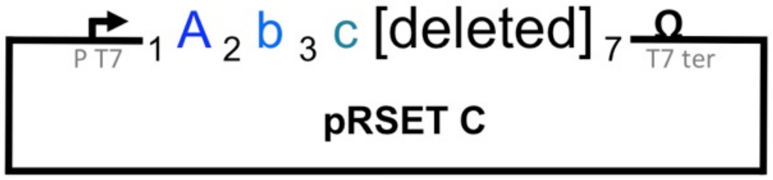

A PZA p56 synthetic ORF
b *mop* intergenic sequence [*E.coli*]
c 10 x His tag
[F HHV1 UDG ORF] deleted to XhoI

1 NdeI
2 PstI
3 NsiI
7 HindIII

Schematic of the bi-cistronic expression construct pRSCΔU56n – with summarised detail of the p56 cassette.

Supplementary information: Part 2 – Results

SDS-PAGE gels for StrepTag II tagged HHV-1 UDG affinity chromatography, following co-expression with p56 wild-type and mutant proteins.

Lanes for each named sample set are as follows (marker lane divides complete sets):

1) pre-induction
2) cell harvest post induction + 16 hours
3) clarified supernatant fraction
4) StrepTactin column flow-through fraction
5) desthiobiotin eluted fraction

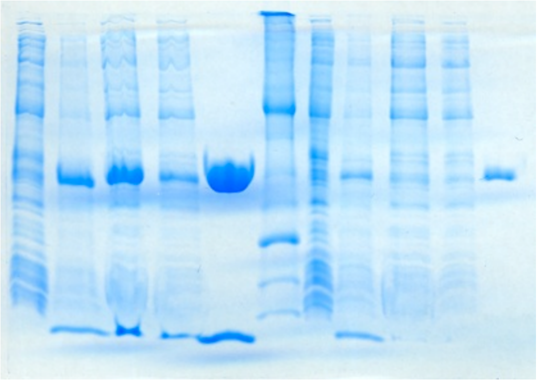	LHS = HHV-1 UDG + p56 [wt]<br><br>Marker lane = Benchmark™ Protein Ladder (Invitrogen)<br><br>RHS = HHV-1 UDG + p56 [E37D/Y40N]
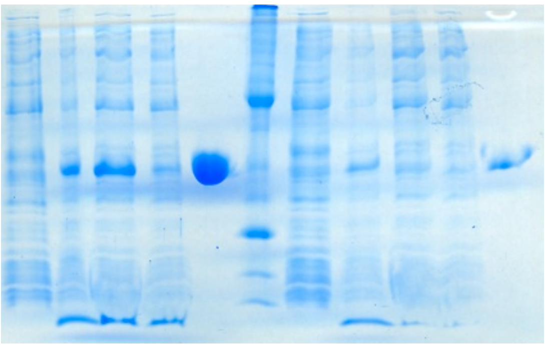	LHS = HHV-1 UDG + p56 [E37D]<br><br>Marker lane = Benchmark™ Protein Ladder (Invitrogen)<br><br>RHS = HHV-1 UDG + p56 [Y40N]

Gel filtration migration volumes, + traces for proteins and selected mixtures in this study: 1/2

L1 – T = 11.25-14.0, P = 12.4

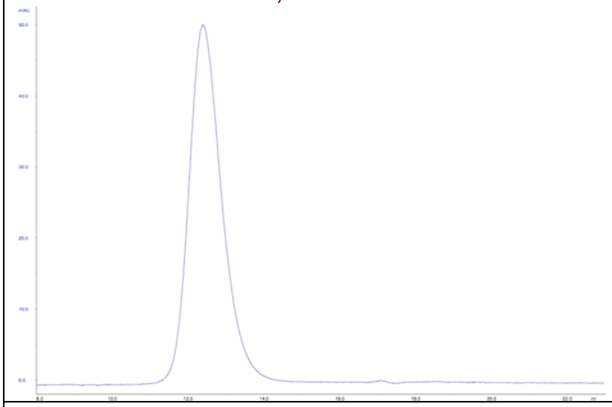

R1 – T = 11.8-14.5, P = 12.7

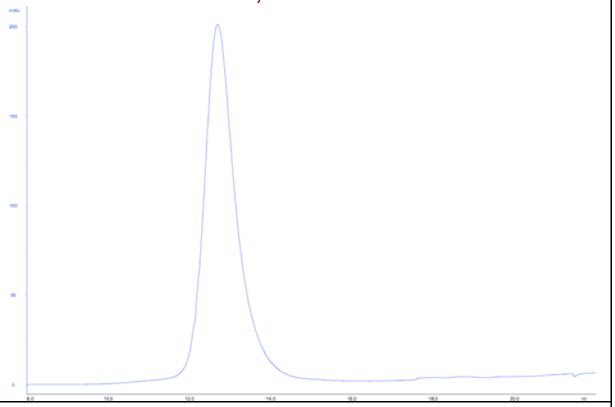

L2 – T = 11.9-14.5, P = 12.6

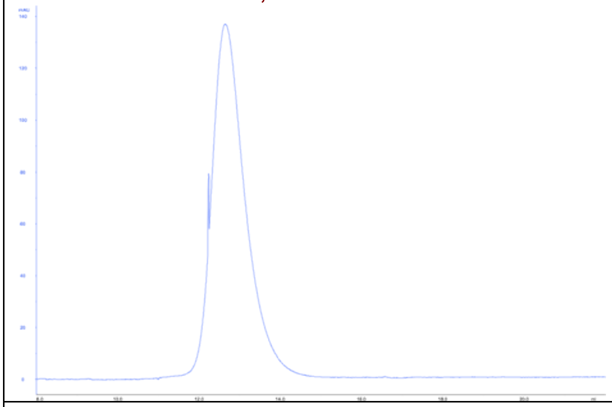

R2 (= L2)

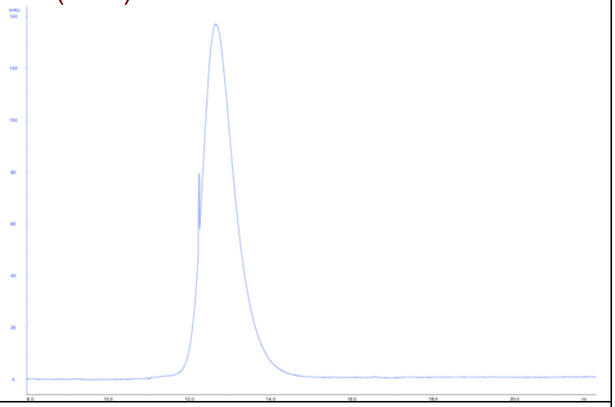

L3 – T = 10.3-12.3, P = 11.2

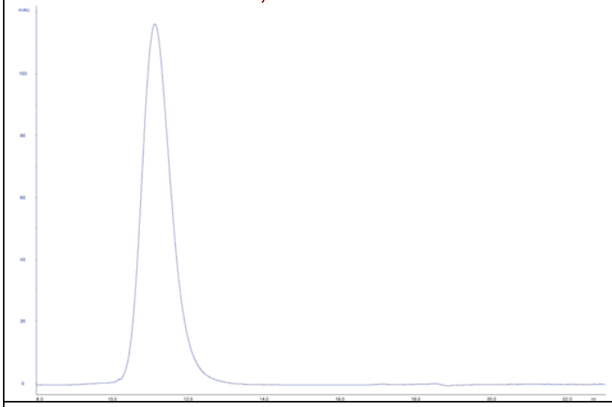

R3 – T = 11.9-14.75, P = 12.8

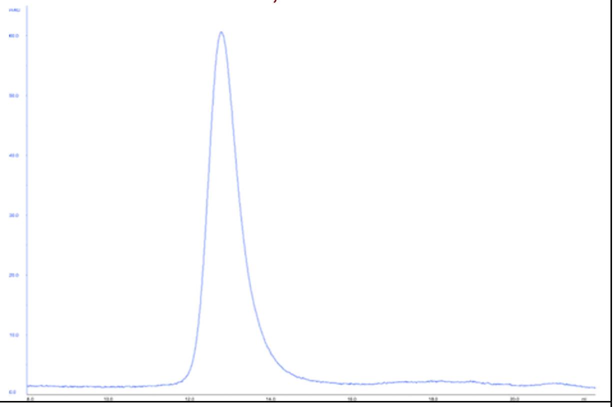

L4 – T = 10.8-14.25, $P_{1,2}$ = 11.4, 12.55

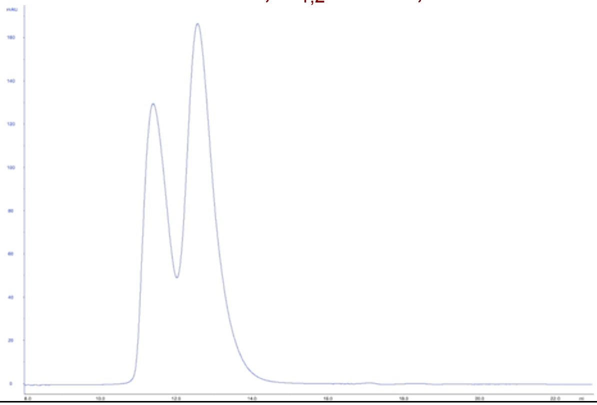

Key: T = peak[start-end], P = peak[apex]

L	R
1) HHV-1 UDG (apo)	1) *E.coli* MUG (apo)
2) p56 WT (apo)	2) p56 WT (apo)
3) UDG + p56 WT	3) MUG + p56 WT
4) UDG + p56 E37D	

Gel filtration migration volumes, + traces for proteins and selected mixtures in this study: 2/2

L1 – T = 11.9-14.4, P = 12.6

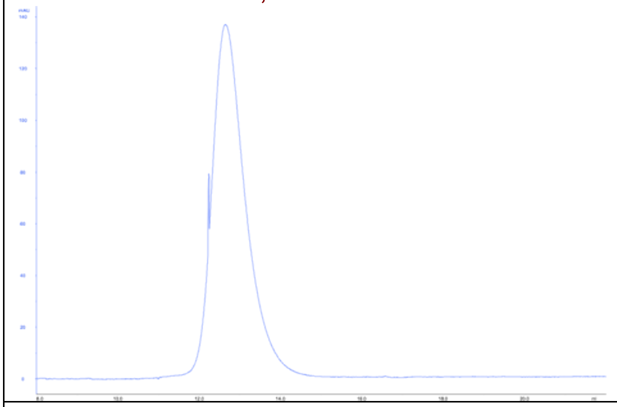

R1 – T = 11.8-14.25, P = 12.6

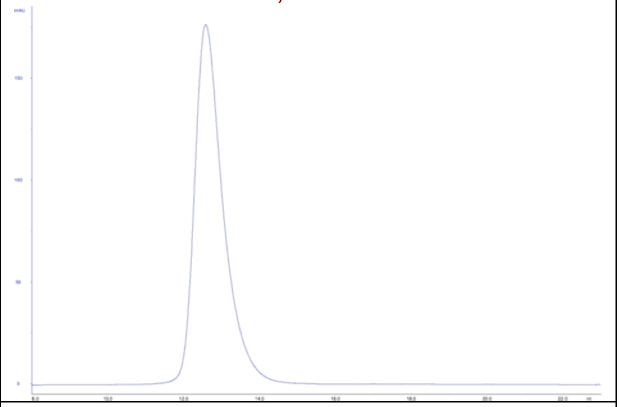

L2 – T = 10.75-15.0, $P_{1,2,3}$ = 11.4, 12.4, 12.9

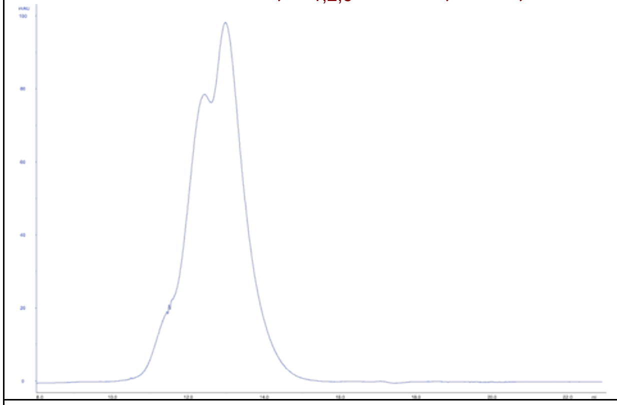

R2 – T = 10.75-14.75, $P_{1,2}$ = 11.5, 13.25

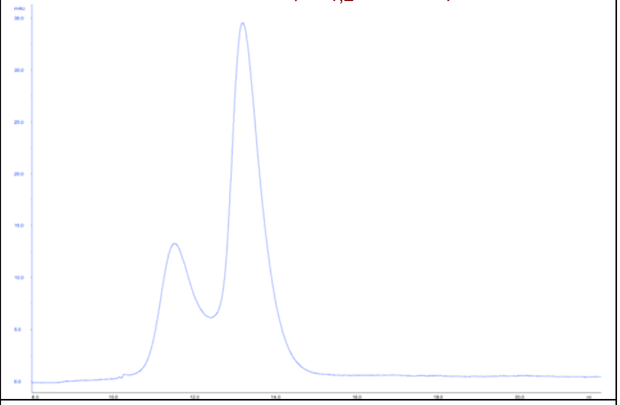

L3 – T = 10.75-15.0, $P_{1,2,3}$ = 11.4, 12.4, 12.9

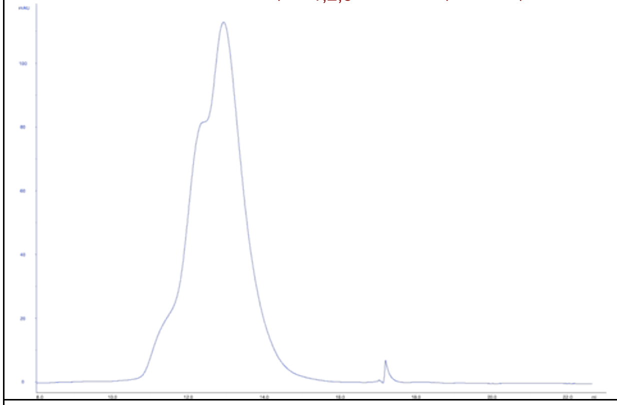

R3 – T = 10.75-14.75, $P_{1,2}$ = 11.5, 13.0

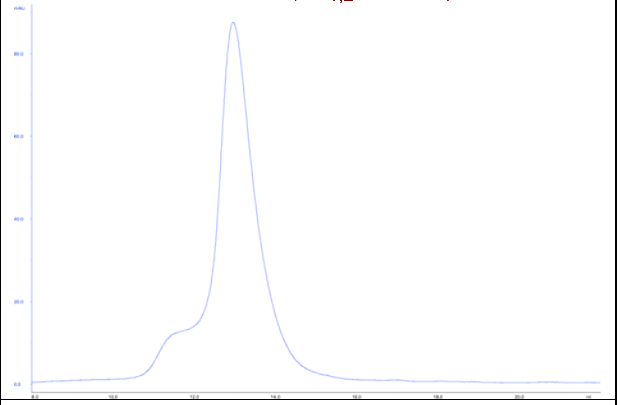

L4 – T = 11.25-14.0, P = 12.4

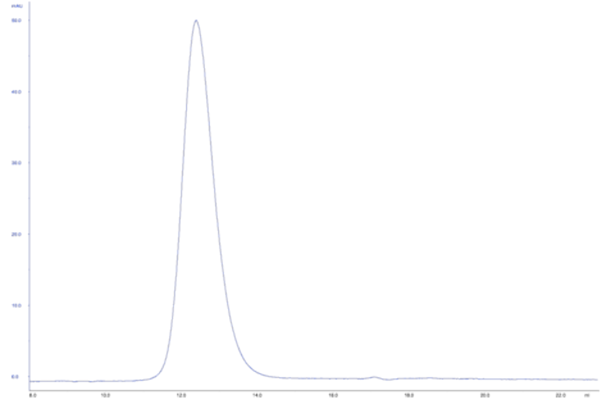

Key: T = peak[start-end], P = peak[apex]

L	R
p56 WT (apo)	p56 [E37D] (apo)
UDG mix p56 [Y40N]	p56 [Y40N] (apo)
UDG mix p56 [E37D/Y40N]	p56 [E37D/Y40N] (apo)
HHV-1 UDG (apo)	

SDS-PAGE gels for gel filtration migration experiments

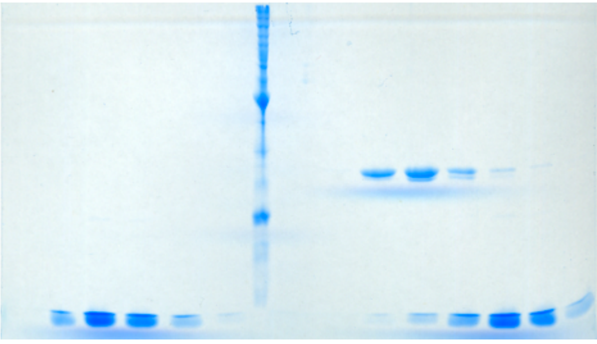	LHS = p56 [E37D] peak fractions<br><br>Marker lane = Benchmark™ Protein Ladder (Invitrogen)<br><br>RHS = HHV-1 UDG + p56 [E37D] peak fractions
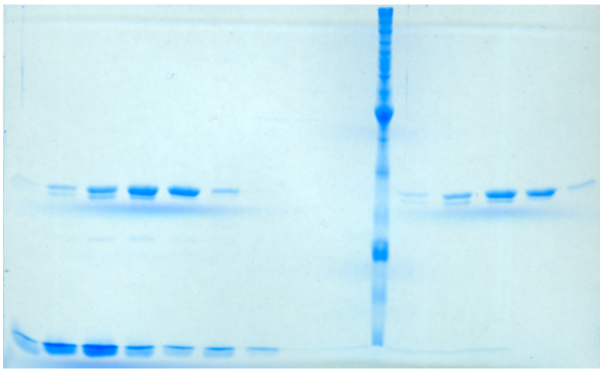	LHS = HHV-1 UDG + p56 [Y40N] peak fractions<br><br>Marker lane = Benchmark™ Protein Ladder (Invitrogen)<br><br>RHS = HHV-1 UDG peak fractions
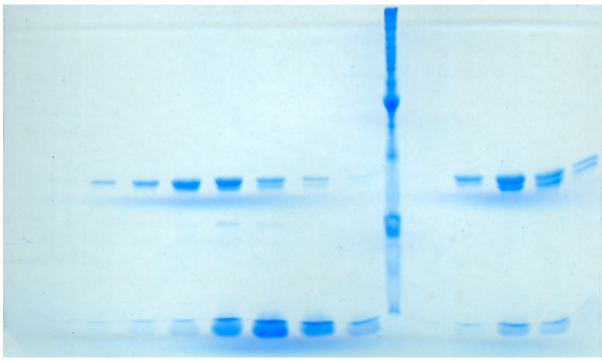	LHS = HHV-1 UDG + p56 [E37D/Y40N] peak fractions<br><br>Marker lane = Benchmark™ Protein Ladder (Invitrogen)<br><br>RHS = HHV-1 UDG + p56 [wt] peak fractions
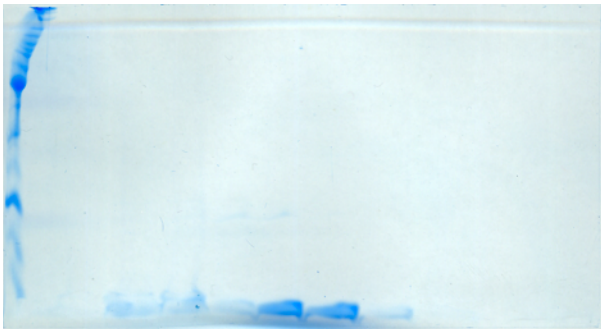	Marker lane = Benchmark™ Protein Ladder (Invitrogen)<br><br>RHS = p56 [Y40N] peak fractions
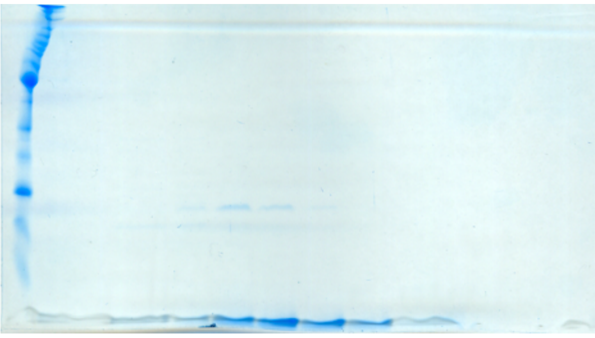	Marker lane = Benchmark™ Protein Ladder (Invitrogen)<br><br>RHS = p56 [E37D/Y40N] peak fractions

Visual U-DNA attrition assay [HHV-1 UDG activity], 1% (w/v) agarose gel.

C1	C2	C3	C4	1	2	3	4	5	6	7	8
ctrl	0.36	3.6	3.6	60	60	7.2	72	360	720	1300	M

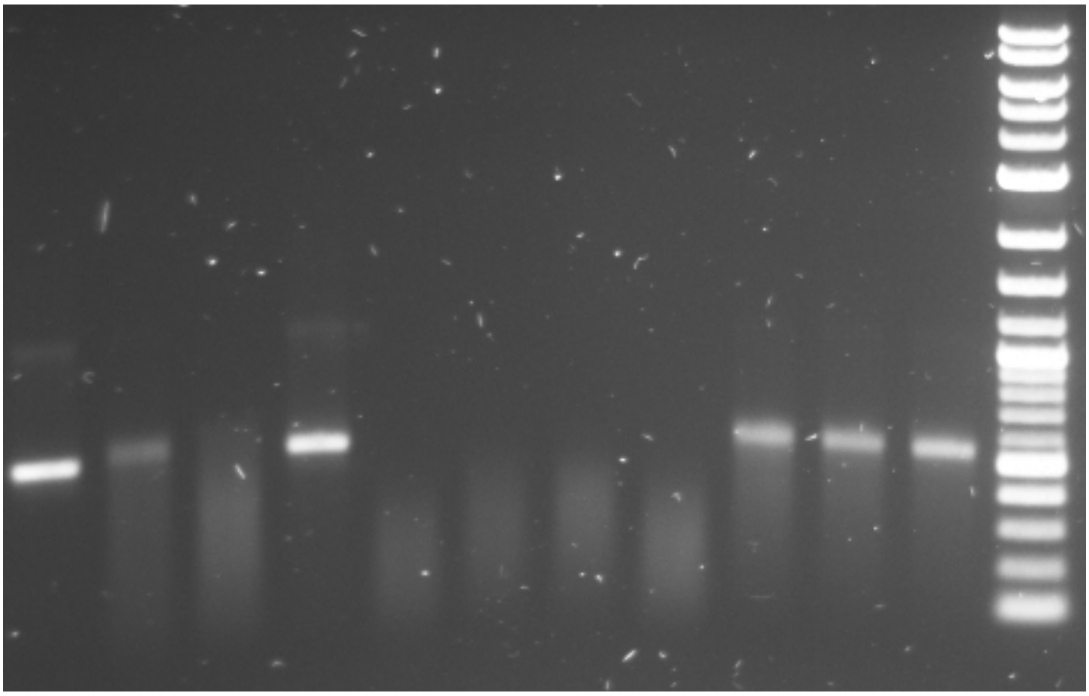

All reactions, including the leftmost control lane, contained 1x NEB buffer 3, Endonuclease IV [also referred to as Nfo] (5 units), and substrate DNA (~500 bp) in which all thymidine is supplanted by deoxyuridine. The DNA concentration for all reactions was set so that it matched the intensity of the nearest sized marker band [NEB 2-log DNA ladder, manufacturer's recommended mass loading] in lane 8 labelled "M". All reactions, including the leftmost control lane were incubated at 37 °C for 30 minutes.
LHS: control lanes (1) DNA substrate only; (2) HHV-1 UDG at 0.36, and (3) 3.6 pmol; (4) HHV-1 UDG and wild-type p56 pre-mixed for 5 minutes in equimolar ratio at 3.6 pmol each. Numbered lanes [top row numbering] are mutant p56 proteins (1 – E37D/Y40N, 2 – Y40N, 3-7 – E37D) added in the pmol quantities indicated [bottom row numbering] to 3.6 pmol HHV-1 UDG 5 minutes prior to substrate addition. Quantities of E37D spanning the range 72 pmol (non-inhibitory) to 360 pmol (an inhibitory effect) were assessed on a separate gel. Other quantities of the Y40N, and E37D/Y40N mutants were assessed on a separate gel.

Visual U-DNA attrition assay [HsSMUG activity], 1% (w/v) agarose gel.

M 1 2 3 4

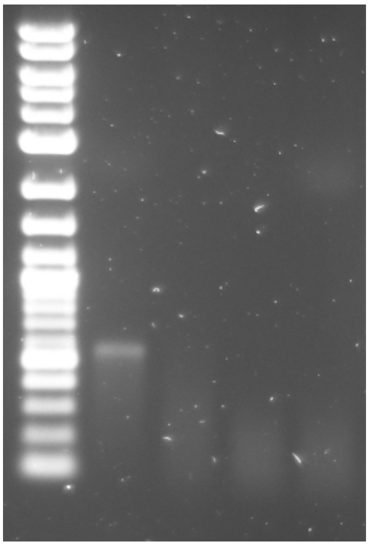

Lane M = NEB 2-log DNA ladder, manufacturer's recommended mass loading.

Reactions (37 °C for 30 minutes; HHV-1 UDG = 1 x NEB buffer 3; HsSMUG = 1 x NEB buffer 1 + 1% BSA), 5 units Nfo included in all reactions.

Lane 1 = 3.6 pmol HHV-1 UDG + 360 pmol p56 [E37D] (mixed 5 minutes prior to substrate addition and incubation).

Lanes 2 to 4 = HsSMUG (1 unit):

2 = HsSMUG only added

3 = HsSMUG + 70 pmol p56 wild-type (5 minute pre-mix)

4 = HsSMUG + 1.3 nmol p56 [E37D] (5 minute pre-mix)

Visual U-DNA attrition assay [HHV-1 UDG activity], 1% (w/v) agarose gel.

C1	C2	C3	1	2	3	4	5	6	7	8	9
ctrl	3.6	3.6	M	250	125	1350	370	185	92.5	185	92.5

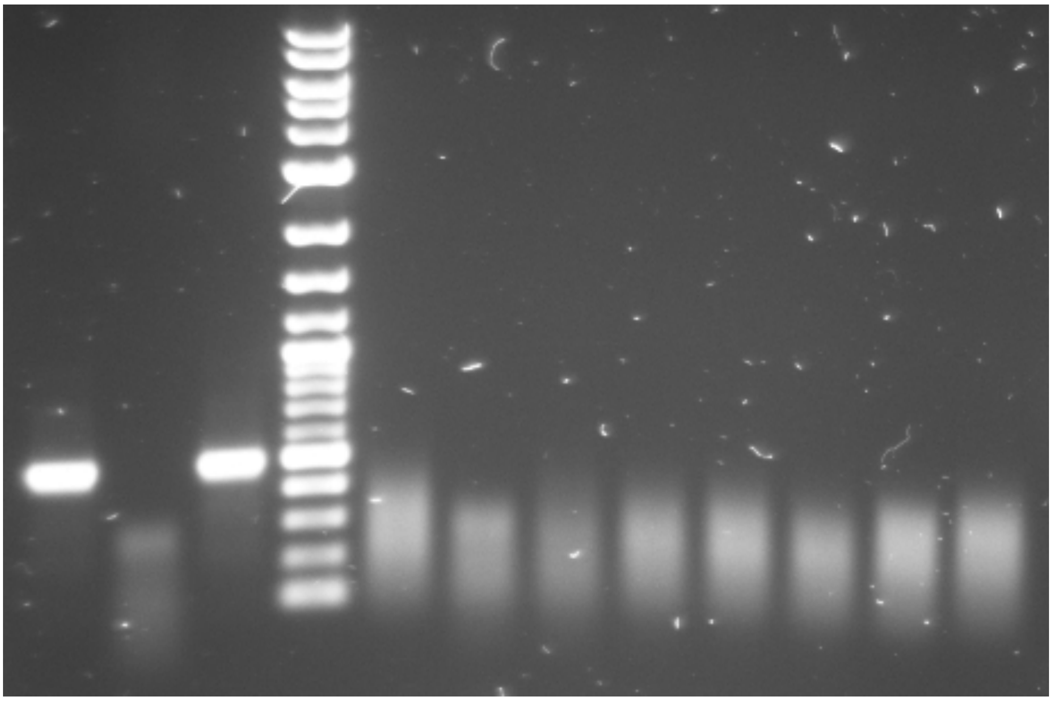

Reaction conditions as described previously.

LHS: control lanes (1) DNA substrate only; (2) HHV-1 UDG at 3.6 pmol + 5 μg BSA; (3) HHV-1 UDG and wild-type p56 pre-mixed for 5 minutes in equimolar ratio at 3.6 pmol each. Numbered lanes [top row numbering] are mutant p56 proteins (2-3 – E37D/Y40N, 4-7 – Y40N, 8-9 – E37D) added in the pmol quantities indicated [bottom row numbering] to 3.6 pmol HHV-1 UDG 5 minutes prior to substrate addition.

Visual U-DNA attrition assay [HHV-1 UDG activity] Nfo$^-$ control, 1% (w/v) agarose gel.

C1 C2 C3 1	
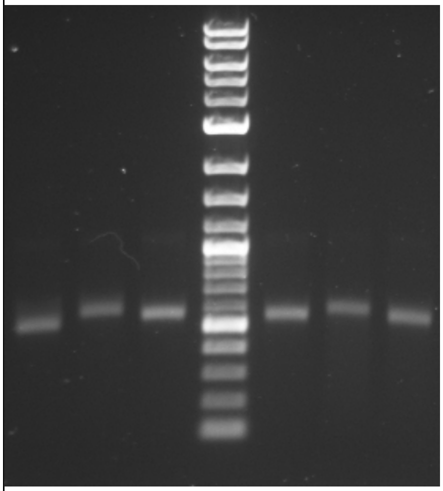	Reactions 37 °C for 30 minutes in 1 x NEB buffer 3, Nfo not added.<br>3.6 pmol HHV-1 UDG added to 1,2,5,6,7.<br>p56 proteins added 5 minutes prior to substrate addition and incubation.<br>Lane 1 = HHV-1 UDG only added.<br>Lane 2 = 360 pmol p56 [E37D] added.<br>Lane 3 = DNA only, no proteins added.<br>Lane 4 = NEB 2-log DNA ladder, manufacturer's recommended mass loading.<br>Lane 5 = 3.6 pmol p56 wild-type added.<br>Lane 6 = 250 pmol p56 [E37D/Y40N] added.<br>Lane 7 = 370 pmol p56 [Y40N] added.

Supplementary information: Part 3 – Figures and Tables

Supplementary figure 1

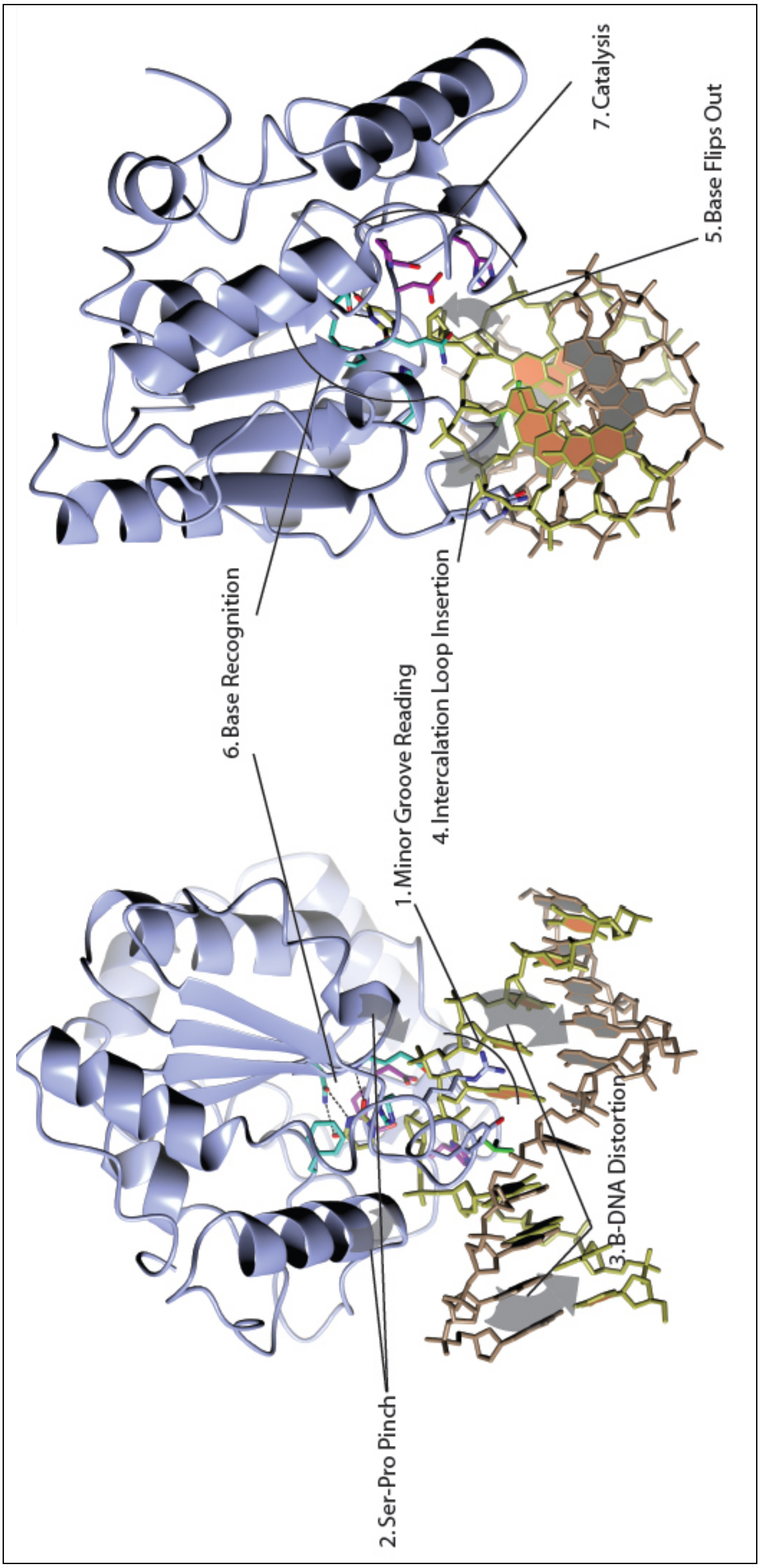

Representation of the catalytic features of family 1 UDG, with reference to the structure 1SSP, Human UDG in complex with a DNA oligomer. Features are referred to throughout the text of the article.

Supplementary figure 2

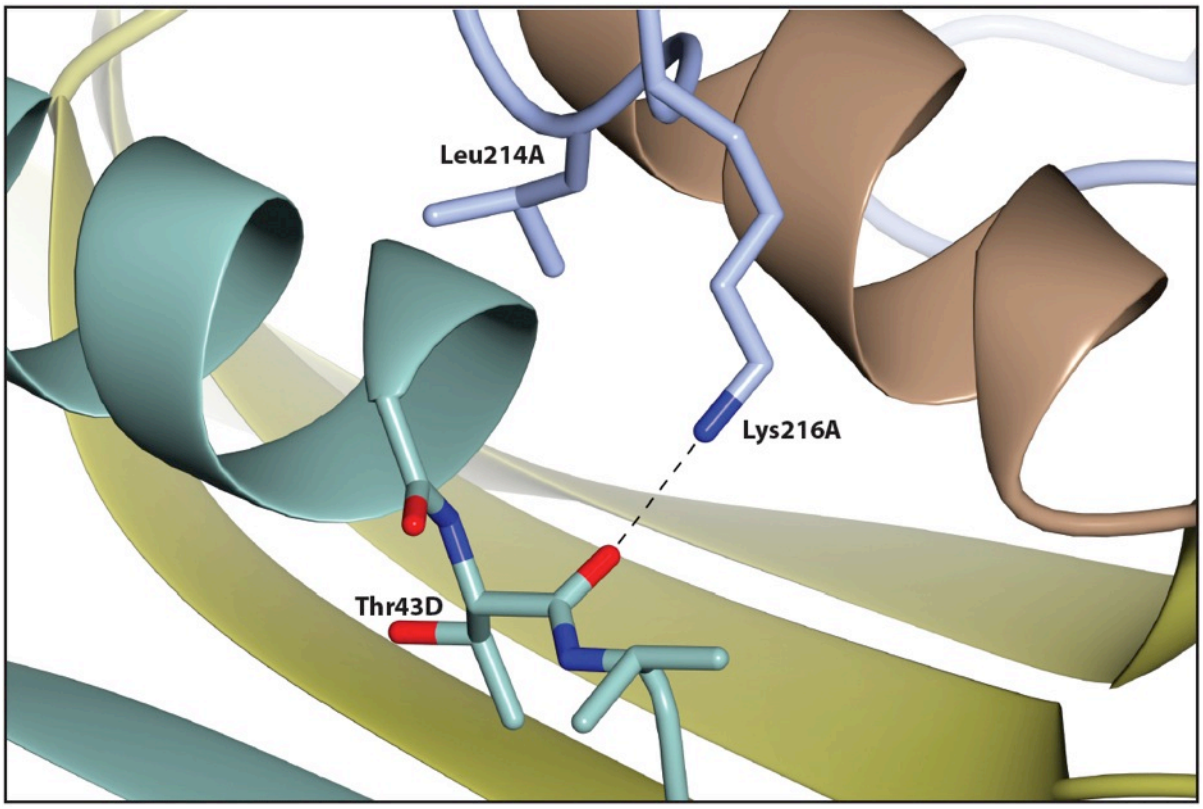

Contacts from the p56 helix not involved in blockade of the UDG DNA binding cleft are limited to the hydrophobic trap, and the p56 Thr43 (molecule D) interaction with HHV-1 UDG Lys216 (molecule A).

Supplementary Figure 3

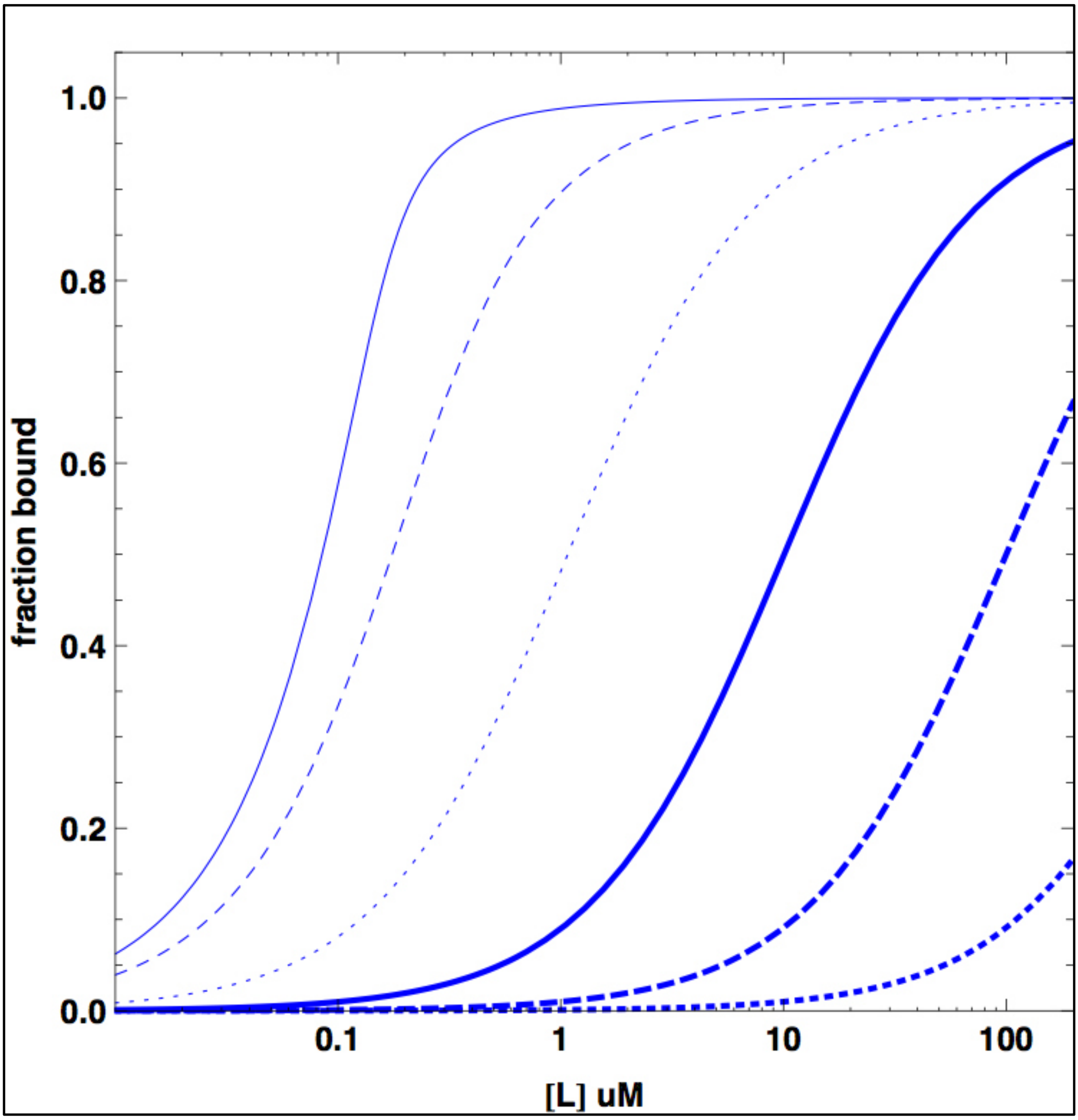

Simulation of percentage of productively bound p56 [E37D] under conditions of assay with HHV-1 UDG. At 100:1 molar ratio, around 10% of this mutant form of the inhibitor might reside in complex with the enzyme. The rates of association and dissociation cannot be taken into consideration, but it is clear from the assay data that DNA is subject to attrition albeit greatly reduced.

Supplementary Table 1

DNA – Human UDG Interactions	ugi – Human UDG Interactions	p56 – HHV-1 UDG Interactions
**DNA Backbone**		
**PO_4 -1**		
His148 Nε2 – PO_4 -1	His148 Nε2 – Ser21 Oγ	His92 Nε2 – Glu26 Oε2
	His148 N – Gln19 O	
	Tyr147 Oη – Glu20 Oε2 via water	
**PO_4 0**		
Gln152 Nε2 – PO_4 0 via water	Gln152 Nε2 – Gln19 O	Gln95 Nε2 – Asn42 O via water
Ser169 NH – PO_4 0	Ser169 NH – Glu20 Oε1	Ser112 NH – Gly41 O
Ser169 Oγ – PO_4 0	Ser169 Oγ – Glu20 Oε2	Ser112 Oγ – Asn42 Oδ1 via water
		Ser112 Oγ – Val38 O via water
**PO_4 +1**		
Gln144 Oε2 – PO_4 +1	Gln144 Nε2 – Leu23 O	Gln87 Nε2 – Asp35 Oδ1
	Gln144 Oε2 – Leu23 NH	
Ser270 Oγ – PO_4 +1	Ser270 Oγ – Thr45 Oγ1	Ser212 Oγ – Glu37 O
	Ser270 Oγ – Glu20 Oε2	
**PO_4 +2**		
Ser247 NH – PO_4 +2	Ser247 NH – Gln28 Oε1	
	Ser247NH – Leu	
His268 NH – PO_4 +2	His268 N Gln28 Oε1 via water	
Ser273 Oγ – PO_4 +2		Ser215 Oγ – Glu37 Oε1 via water
		His191 Nδ1- Asp35 Oδ1
		His191 Nδ1 – Ser32 Oγ
	Tyr248 N – Leu23 O via water	
**Minor Groove Reading Head**		
Arg276 Nε – Base +1 via water		Lys216 Nζ – Thr43 O [molecule D]
**Uracil Recognition**		
His268 Nε2 – Uracil N1	His268 N – Gln28 via water	
Gln144 NH – UracilO2		
**Catalytic Site**		
Asp145 Oδ1 – Catalytic Water	Asp145 Oδ2 – Ser21 O via water	Asp88 Oδ1 – Asn42 Oδ1 via water

A comparison of interactions between UDG and molecules discussed in the text: column 1 – Human UDG and dsDNA (sourced from PDB entry 1SSP); column 2 – Human UDG and ugi (sourced from PDB entry 1UGH); column 3 – HHV-1 UDG and p56 [phage PZA] (as observed in the present study). Phosphate positions are 0 for the nucleotide at the catalytic site, and -1, +1, and +2 for the flanking nucleotides. Comparisons are also elaborated in the main article text.
